# AddaGCN: Spatial transcriptomics deconvolution using graph convolutional networks with adversarial discriminative domain adaptation

**DOI:** 10.1371/journal.pcbi.1014609

**Published:** 2026-08-05

**Authors:** Shuzhen Ding, Zhou Yu, Jingsi Ming

**Affiliations:** 1 KLATASDS-MOE, School of Statistics, East China Normal University, Shanghai, China; 2 College of Mathematics and Systems Sciences, Xinjiang University, Urumqi, China; 3 Academy of Statistics and Interdisciplinary Sciences, East China Normal University, Shanghai, China; University of Western Ontario: Western University, CANADA

## Abstract

The rapid advancement of spatial transcriptomics has substantially improved our understanding of the spatial architecture and gene expression heterogeneity within tissues. However, many spatial transcriptomics techniques can not reach single-cell resolution, instead measuring gene expression profiles from mixtures of potentially heterogeneous cell types. Here we propose AddaGCN, a robust deconvolution method to infer cell type composition from spatial transcriptomic data. AddaGCN leverages graph convolutional networks to incorporate spatial information and adopts an adversarial discriminative domain adaptation approach to mitigate batch effects between spatial and single-cell reference data. Comprehensive analyses of real data generated by diverse technology platforms demonstrate AddaGCN’s superior performance and robustness in cell-type deconvolution compared to other methods. These analyses further reveal AddaGCN’s potential to uncover spatiotemporal changes during tissue development and to characterize the tumor microenvironment.

## Author summary

This article mainly discusses the challenges of low-resolution spatial transcriptomics and presents a novel computational method, AddaGCN, designed to accurately infer cell type composition through advanced neural network techniques. Our approach models both spatial and expression relationships among cells using graph neural networks, employs adversarial training to align synthetic and real data distributions and reduce batch effects, and dynamically adapts representations during training to better resolve complex cellular colocalization patterns. When evaluated across multiple ST platforms, AddaGCN consistently outperforms existing methods in accuracy and robustness. AddaGCN also demonstrates strong performance in several biologically relevant applications, including precise identification of pancreatic and tumor subtypes, characterization of spatiotemporal developmental dynamics, and mapping of cell–cell interactions in the tumor microenvironment. AddaGCN provides a reliable and versatile computational framework for decoding tissue architecture from spatial transcriptomics data.

## Introduction

Spatial transcriptomics (ST) has revolutionized biological research by enabling the mapping of gene expression profiles within tissue architectures, thereby preserving crucial spatial context that is absent in single-cell RNA sequencing (scRNA-seq) [1–4]. This technology provides unprecedented insights into cellular heterogeneity, tissue organization, and microenvironmental interactions, which are pivotal for understanding developmental processes, disease mechanisms, and therapeutic responses [5–7]. However, one of the major limitations of current ST platforms, such as 10x Visium [8], is their limited spatial resolution. Each measured spot typically captures transcriptional signals from multiple adjacent cells, resulting in composite expression profiles that mask cell-type-specific signatures. Consequently, the inability to disentangle cell-type-specific expression patterns within these mixed spots hinders biological interpretation and limits the utility of ST in dissecting complex tissue architectures [9,10].

To address this challenge, computational deconvolution methods have emerged as essential tools for inferring the cell-type proportions within each spot. These methods are fundamentally based on the integration of scRNA-seq data, which offers transcriptional profiles of distinct cell types [11–14]. For example, SPOTlight [15] builds upon a non-negative matrix factorization regression algorithm initialized with scRNA-seq data. Cell2location [16] is a Bayesian model that decomposes the spatial transcriptomics data into reference cell type signatures constructed from scRNA-seq data. Tangram [17] is a deep-learning framework which maps single-cell data to spatial data and predicts cellular composition. Other deep learning-based methods, such as CellDART [18] and Spoint [19], construct pseudo-ST data with reference scRNA-seq data and use neural network to integrate it with real-ST data for predicting cell-type proportions.

However, scRNA-seq alone is inadequate for achieving reliable spatial deconvolution. Given that neighboring spots are more likely to have similar cellular compositions, spatial coordinates impose topological constraints that can enhance the accuracy of cell-type proportion estimates. This highlights the necessity of incorporating spatial information into deconvolution frameworks [20,21]. Graph convolutional networks (GCNs) [22] show significant potential in enhancing model performance by leveraging the inherent topological information within the data. Incorporating GCNs into the model enables more efficient use of spatial location information. Some deep learning methods based on pseudo-ST data generation have integrated GCNs, including DSTG [23], SD2 [24] and STdGCN [25]. However, the performance of these methods remains constrained by the stochastic nature of pseudo-ST generation, the complexity of cellular colocalization, and platform-specific biases between spatial and single-cell data. Adversarial domain adaptation provides a principled framework for aligning feature distributions across different data platforms without requiring paired samples, making it a natural choice for mitigating such cross-domain discrepancies while preserving cell-type-specific signals. Therefore, it is essential to develop robust and adaptable deconvolution frameworks that fully realize the potential of ST in spatially resolved systems biology.

In this study, we propose the adversarial discriminative domain adaptation graph convolutional network (AddaGCN) to infer the cell composition of ST data. Our model employs GCNs to incorporate spatial information and capture the correlations between pseudo and real-ST data. Additionally, we adopt an adversarial domain adaptation training approach to reduce the batch effects between pseudo and real-ST data, enabling the learned proportion inference model in the pseudo-ST domain to be applicable in the real-ST domain. To evaluate the performance of AddaGCN, we conducted analyses on ST datasets from various platforms. The results consistently demonstrate the superiority of AddaGCN over other competing methods, highlighting its advantages in terms of accuracy and robustness for the cell-type decomposition of ST data.

## Results

### Overview of AddaGCN

**Fig 1:**
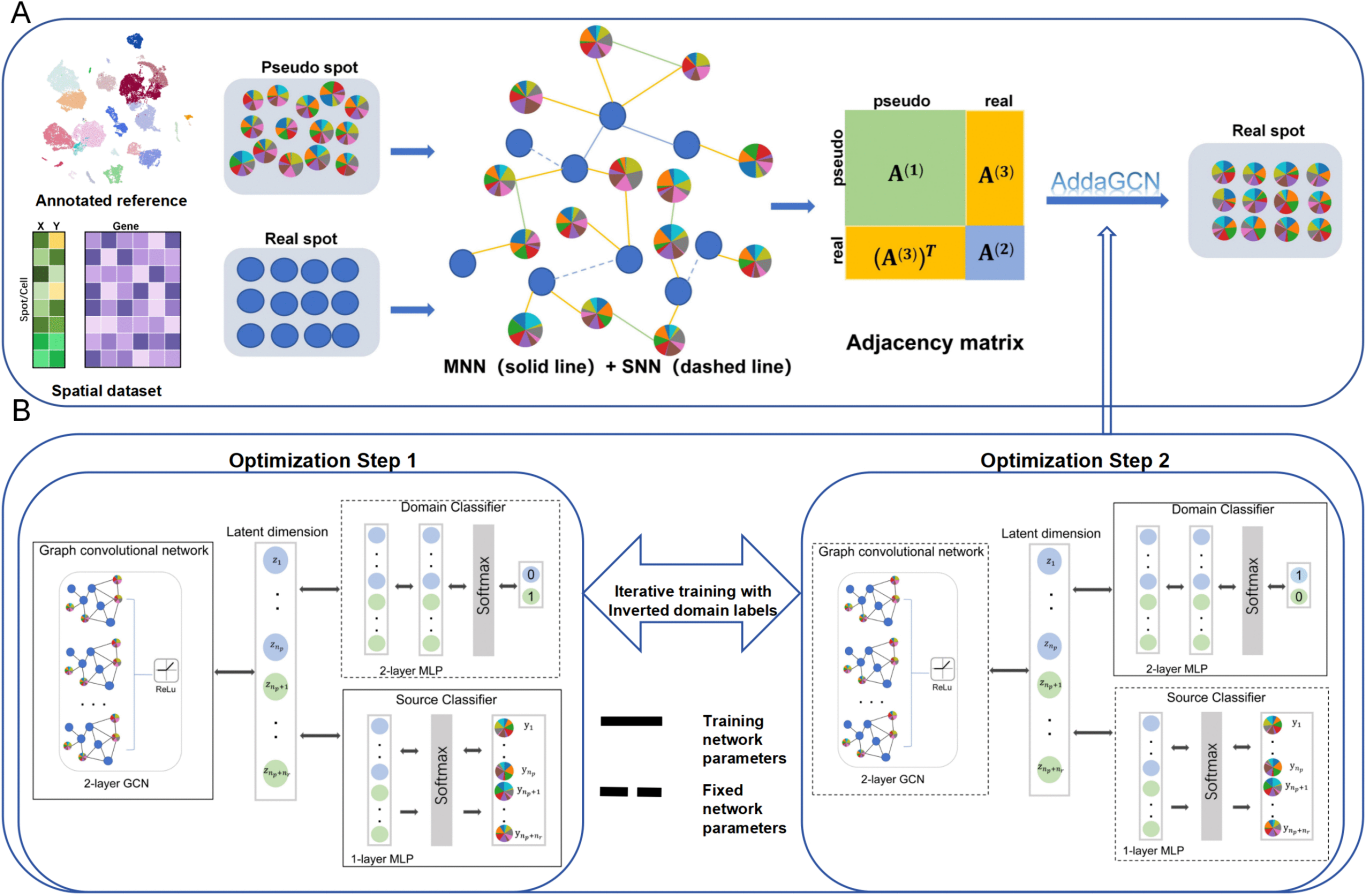
Overview of the AddaGCN framework. **(A)** First, we use the annotated scRNA-seq reference data to generate a pseudo-spot pool. Then, a link graph is constructed among pseudo and real-spots based on their transcriptional similarity and spatial locations. Finally, AddaGCN is applied to infer the cell-type composition for real-ST spots. **(B)** AddaGCN consists of a GCN feature extractor, a source classifier, and a domain classifier. The GCN feature extractor learns robust graph-based representations of spots. The source classifier predicts cell-type composition for each spot. The domain classifier distinguishes between pseudo and real-spots. Training involves two iterative optimization steps. In step 1, the GCN feature extractor and source classifier are trained while the domain classifier is fixed. In step 2, with inverted domain labels, the domain classifier is trained while the GCN feature extractor and source classifier are fixed. Finally, the trained AddaGCN model is capable of predicting cell-type proportions for real-ST data.

AddaGCN is designed for cell-type deconvolution in ST data, leveraging scRNA-seq data with known cell-type labels as a reference (Fig 1A). To capitalize on the cell-type specific gene profile information in scRNA-seq, pseudo-spots with known cell type compositions are generated to mimic the characteristics of real-ST data. To establish connections between pseudo-spots and real-spots, a link graph is constructed based on the mutual nearest neighbors (MNN) and the spatial neighbor network (SNN). The MNN graph is established according to the similarity of gene expression patterns between and among pseudo-spots and real-spots. The SNN graph is constructed based on the spatial locations of real-spots to incorporate spatial information. By applying AddaGCN with the combined adjacency matrix, the cell type compositions of real-spots can be inferred.

AddaGCN consists of three components: a GCN feature extractor, a source classifier, and a domain classifier (Fig 1B). The GCN feature extractor aggregates graph signals from node neighborhoods, facilitating the learning of robust graph-based representations. The source classifier predicts the cell type composition within each spot and can be trained using pseudo-ST data. However, due to the inherent technical biases between scRNA-seq and ST data, as well as the randomness in pseudo-spot generation, batch effects may occur between pseudo and real-ST data. This makes it difficult to transfer the source classifier for the analysis of real-ST data. To address this issue, AddaGCN adopts an enhanced adversarial discriminative domain adaptation (ADDA) approach [26]. By leveraging the domain classifier that distinguishes between pseudo-spots and real-spots, we iteratively train the domain classifier and source classifier with inverted domain labels. This adversarial training strategy enables the GCN feature extractor to align feature distributions of two domains, and the source classifier to provide accurate and robust prediction of cell-type compositions for real-ST data.

### Benchmark evaluation

To evaluate the performance of AddaGCN, we utilized two single-cell resolution ST datasets generated using seqFISH+ [1] and Xenium [11] platforms to simulate ST data by gridding and aggregating single cells (Text A in S1 Appendix). We compared AddaGCN with ten existing deconvolution methods, including CellDART, STdGCN, DSTG, SD2, Spoint, Tangram, SpatialDecon [27], Cell2location, DOT [28] and SPOTlight on the simulated datasets. To quantitatively evaluate the accuracy of deconvolution methods, we used four metrics: Pearson correlation coefficient (PCC), structural similarity (SSIM), root mean square error (RMSE) and Jensen-Shannon divergence (JSD), along with an overall average rank (Text B in S1 Appendix).

**Fig 2:**
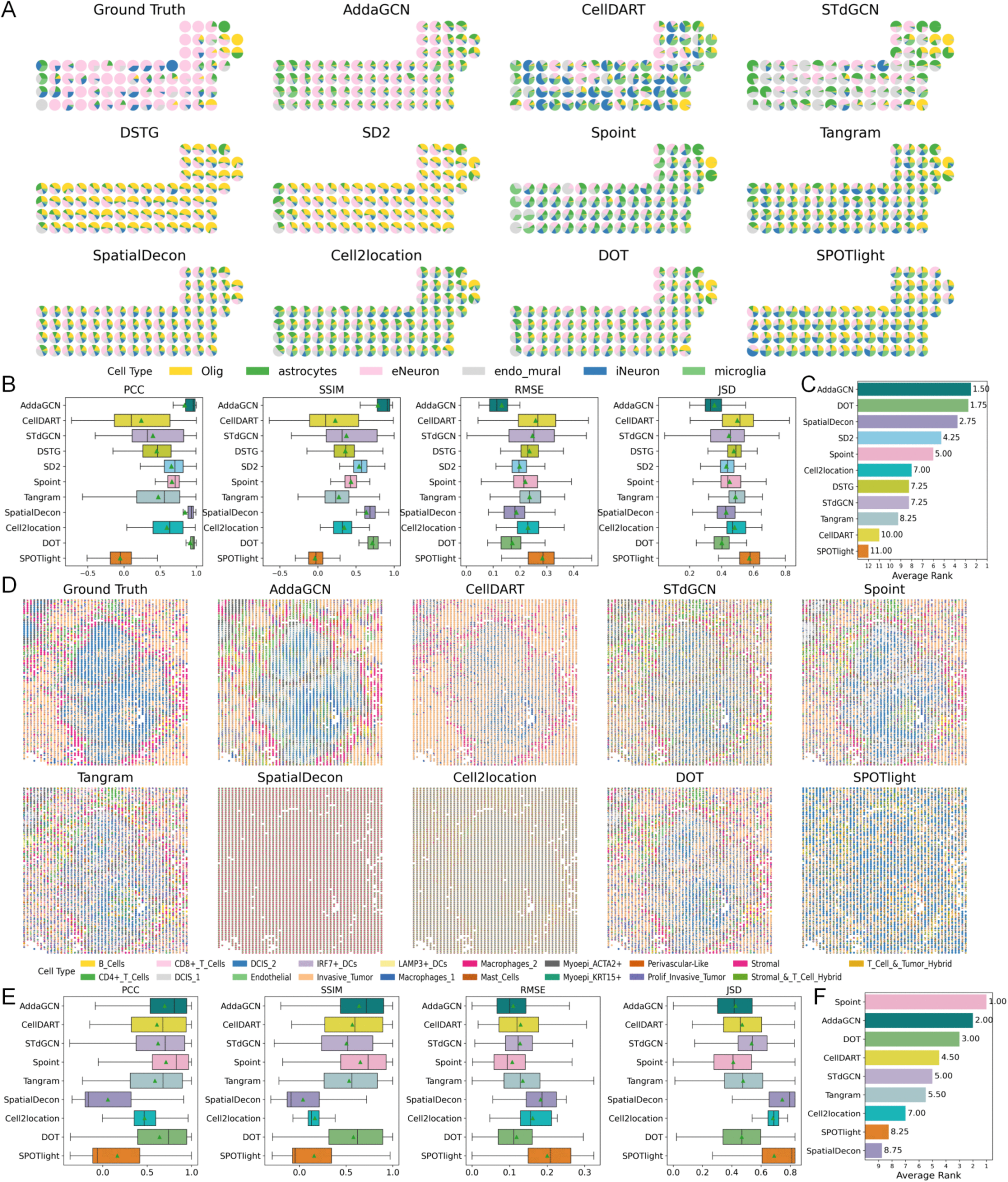
Benchmark evaluation on the seqFISH+ data and the Xenium data. **(A, D)** A spatial scatter pie plot displays the true and the inferred cell-type composition of seqFISH+ data (A) and Xenium data (D) from different deconvolution methods. **(B, E)** Boxplots of PCC, SSIM, RMSE and JSD of each deconvolution method in predicting the cell type distribution of spots. Center line: median; box limits: upper and lower quartiles; whisker: 1.5 × interquartile range; green triangle: mean value; number of predicted spots: 71 for the seqFISH+ data **(B)**; 4237 for the Xenium data **(E)**. Higher PCC and SSIM, lower RMSE and JSD indicate better performance. **(C, F)** Bar plots of average rank (aggregated from PCC, SSIM, RMSE and JSD) of prediction accuracy on the seqFISH+ data (C) and the Xenium data **(F)**.

For the seqFISH+ dataset, we used a publicly available scRNA-seq dataset [29] as the reference for deconvolution. AddaGCN demonstrated the most accurate deconvolution results with respect to the true cell type composition in the slice (Fig 2A). For each cell type, the predicted cell distributions in the slice using AddaGCN were highly consistent with the true distributions (Fig A in S1 Appendix). For example, AddaGCN effectively identified the spatial patterns of excitatory neurons, which represent the predominant cell type in the slice. However, other methods detected a notably lower number of excitatory neurons. Moreover, AddaGCN consistently exhibited the highest PCC and SSIM values, as well as the lowest values and ranges for both JSD and RMSE compared to other methods, achieving the highest overall average rank (Fig 2B and 2C). The superior performance of AddaGCN over other graph-based methods suggests that the improvement is not solely due to graph-based modeling, but also reflects the contribution of the adversarial domain adaptation component. The dual-discriminator adversarial framework in AddaGCN jointly encourages the preservation of cell-type-specific signals while promoting the learning of domain-invariant representations, mitigating cross-platform batch effects beyond conventional graph-based approaches.

Regarding the Xenium data, we selected a small region in the slice and used another region to serve as the single-cell reference (Fig B in S1 Appendix). Since DSTG and SD2 failed to provide valid results on this dataset, we excluded these two methods from our comparison. AddaGCN demonstrated the ability to locate tumor boundaries and distinguish invasive tumor cells from normal endothelial cells (Figs 2D and C in S1 Appendix). Compared with other methods, AddaGCN and Spoint ranked among the top two according to the four metrics and the overall average rank, highlighting their effectiveness in spatially resolved transcriptomic analysis (Fig 2E and 2F). To evaluate whether AddaGCN remains reliable when the scRNA-seq reference differs substantially in cell-type composition from the ST data, we designed five simulation scenarios with varying tumor burdens (31.57% to 1.35%) and pronounced compositional mismatches between reference and spatial regions (Fig D and Table A in S1 Appendix). AddaGCN maintained stable performance across all scenarios, demonstrating robustness to substantial shifts in cell-type proportions.

To further assess the batch-correction capability under realistic cross-study conditions, we evaluated AddaGCN using an independent breast cancer scRNA-seq reference (GSE176078) [30] distinct in study origin, protocol, and platform from the Xenium ST data (Text A in S1 Appendix). Despite the substantial domain discrepancy between the spatial and single-cell datasets, AddaGCN achieves both structural fidelity and cell-type specificity and consistently achieves the best or near-best performance across evaluation metrics and obtained the top overall average ranking among all competing methods (Figs E and F in S1 Appendix). These results demonstrate that the adversarial domain adaptation component effectively aligns feature distributions across independent studies, supporting the robustness of AddaGCN for practical applications where matched references are unavailable.

We benchmarked the computational efficiency of AddaGCN against baseline methods on two Xenium-based simulated datasets of different scales, with each configuration completing within 5–15 minutes on an NVIDIA GeForce RTX 4090 GPU (24GB) (Table B in S1 Appendix). The results demonstrate that AddaGCN achieves a favorable trade-off between computational efficiency and resource consumption among deep learning-based approaches.

AddaGCN comprises several key components, including the MNN-based linking, spatial SNN, the GCN module, and the adversarial domain adaptation mechanism. To evaluate the contribution of each component, we conducted ablation experiments using the simulated seqFISH+ data. It is evident that the adversarial domain adaptation mechanism and the GCN module have the most significant impact on prediction accuracy, followed by spatial SNN and MNN-based linking, which also have substantial impacts and represent the innovation of the AddaGCN algorithm (Fig G in S1 Appendix).

### Human pancreatic ductal adenocarcinoma (PDAC) data

**Fig 3:**
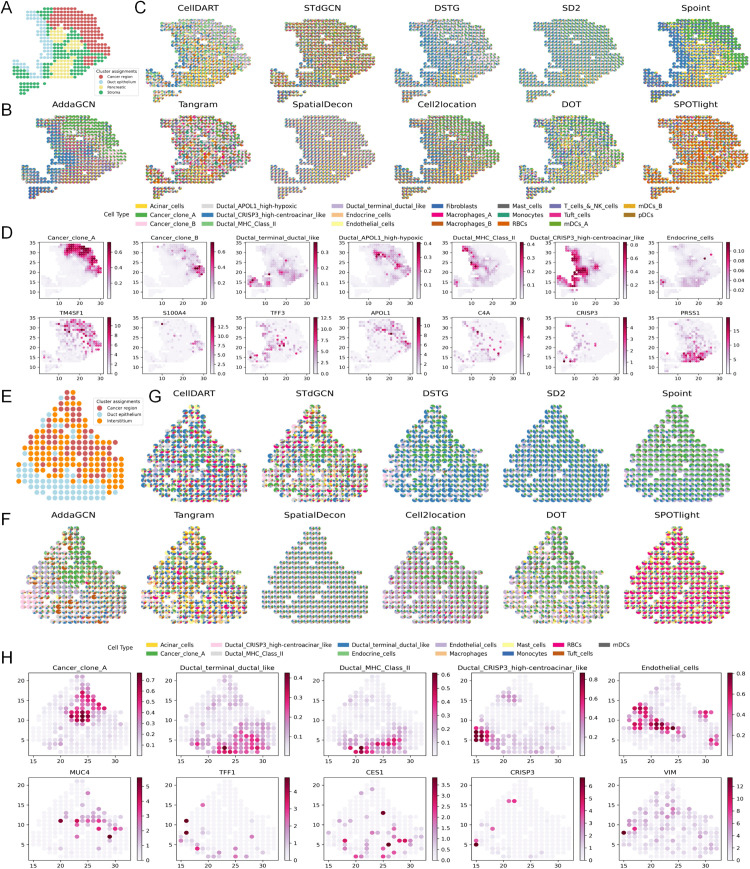
AddaGCN analysis in PDAC data. **(A, E)** PDAC-A slice (A) displays four regions annotated from the original publication [6]. Clustering of the PDAC-B **(E)** ST spots, with colors indicating Leiden cluster assignments (standard Scanpy pipeline). For detailed H&E-stained region annotation results, please refer to Fig 2a (PDAC-A) and Fig 2b (PDAC-B) in the original publication [6]. **(B, F)** A spatial scatter pie plot displays the inferred cell-type composition using AddaGCN for PDAC-A (B) and PDAC-B **(F)**. **(C, G)** A spatial scatter pie plot displays inferred cell-type composition using comparative deconvolution methods for PDAC-A (C) and PDAC-B **(G)**. **(D, H)** Top, the proportions of cell types inferred by AddaGCN for PDAC-A (D) and PDAC-B (H) are displayed. Bottom, the normalized gene expressions of corresponding cell-type specific marker genes are displayed.

To conduct a more comprehensive assessment of the performance of AddaGCN on pathological data, we applied it to pancreatic ductal adenocarcinoma (PDAC) slices from two tumor sections, designated PDAC-A and PDAC-B. We utilized matched scRNA-seq data generated by the inDrop protocol as the reference for deconvoluting the PDAC ST data. The PDAC dataset encompasses various tissue regions, including cancerous, normal pancreatic, ductal, and interstitium areas. These regions were annotated by histologists based on hematoxylin and eosin (H&E) staining [6] (Fig 3A) and leiden cluster assignments (Fig 3E).

For the PDAC-A slice, AddaGCN effectively identified and localized diverse pancreatic and tumor cell types across distinct tissue regions (Fig 3B). Specifically, cancer clone cells were predominantly found within the cancerous regions located in the upper-right corner of the slice; ductal cells clustered at the left edge of the slice; and normal pancreatic cells (e.g., acinar cells and endocrine cells) and interstitium cells (e.g., fibroblasts and endothelial cells) were situated between the ductal and cancer areas. These observations are consistent with the results annotated via H&E staining and brightfield imaging [6] (Fig 3A). In contrast, the deconvolution results of STdGCN, DSTG, SD2, Tangram, SpatialDecon and SPOTlight failed to show an obvious distinction among the four different tissue regions (Fig 3C). Moreover, STdGCN, DOT and SPOTlight encountered difficulties in clearly differentiating between cancerous and non-cancerous regions. The results obtained by AddaGCN also exhibited excellent spatial continuity and consistency with the corresponding marker gene expression patterns (Fig 3D). However, CellDART, STdGCN, Tangram, Cell2location, and SPOTlight displayed highly dispersed distribution patterns (Fig H in S1 Appendix). In terms of cancer clone region detection (Text A in S1 Appendix), AddaGCN demonstrates the advantage of achieving an optimal precision-recall balance with minimal false positives, substantially outperforming methods suffering from either over-prediction (Spoint) or detection failure (DSTG, SD2, SPOTlight) (Fig I and Table C in S1 Appendix).

Furthermore, AddaGCN distinguished the cancerous regions into two sub-regions (Fig 3D). The upper sub-region was primarily enriched with cancer clone A cells, identified by the marker gene TM4SF1, and the lower sub-region was enriched with cancer clone B cells, marked by S100A4. Given that S100A4 is a prognostic marker for early-stage pancreatic cancer, it is implied that the lower cancer sub-region may represent an earlier stage of the disease. Conversely, TM4SF1 is known to play a crucial role in PDAC metastasis and invasion [31–33], suggesting that the upper cancer sub-region may correspond to a later stage with metastatic potential. However, all other methods showed limited discriminative capacity between these two types of cancer clones (Fig H in S1 Appendix).

For the PDAC-B slice, AddaGCN identified cancer clone cells that were preferentially localized to spots in the upper right region, effectively distinguishing them from the interstitium and ductal areas (Fig 3F). Notably, most interstitium cells were located adjacent to cancer clone cells, with some even co-localizing with them, while ductal cells were predominantly concentrated in the lower region spots (Fig 3H). These findings are highly consistent with the previous results from H&E staining [6] and the clustering of the PDAC-B ST spots (Fig 3E). In contrast, alternative methods either had difficulties in identifying cancer regions or lacked the clarity required to distinguish between ductal and interstitium cells (Figs 3G and J in S1 Appendix). Overall, the results from AddaGCN closely align with independent histological annotations, highlighting its capability to accurately identify cell compositions from ST data of tumor tissues.

### Developing human heart spatial transcriptomic dataset

We further demonstrate the capabilities of AddaGCN in exploring the dynamic spatial-temporal changes in cell-type distribution. This exploration is based on three developmental stages of the human embryonic heart, i.e., 4.5-5, 6.5, and 9 post-conception weeks (PCW) [34].

**Fig 4:**
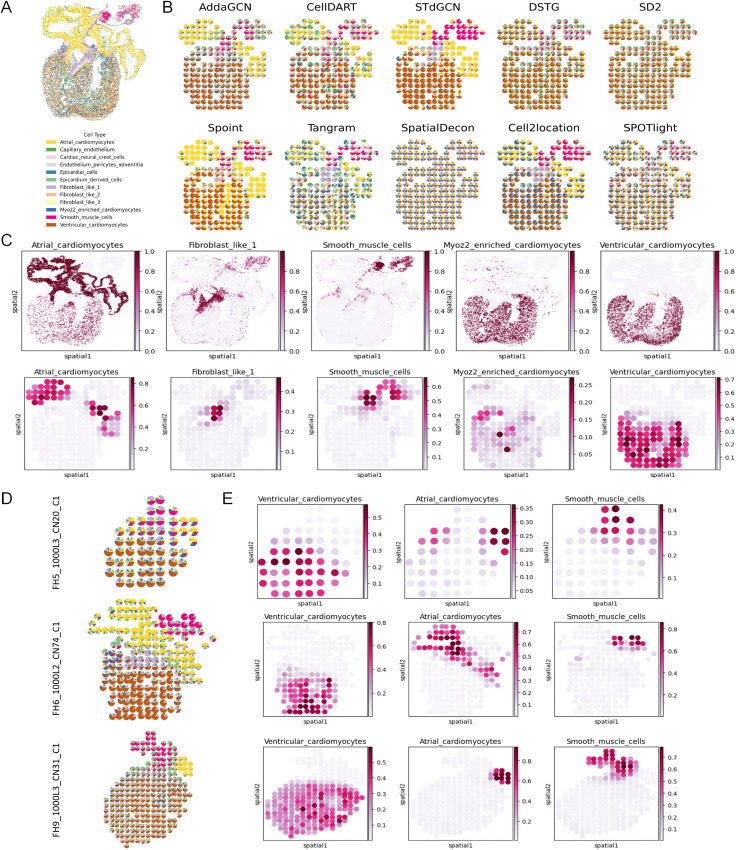
Developing human heart spatial transcriptomic dataset. **(A)** The spatial structure and cell-type distribution of cells identified by ISS in a PCW6.5 heart tissue section. **(B)** The pie plot displays the predicted cell-type proportions of different methods of the PCW6.5 heart tissue section (FH6_1000L2_CN74_D1). **(C)** Top panel: Ground-truth cell type spatial distribution from ISS data at single-cell resolution. Each dot represents one cell colored by its annotated type; Lower panels: Scatter plots display the predicted proportions from the PCW6.5 heart tissue section using AddaGCN. **(D)** The AddaGCN method was applied to infer the distribution of different cell-types across developmental stages. **(E)** The resulting estimates of the spatial distribution of different cell types.

To validate the effectiveness of AddaGCN on this data, we first applied AddaGCN to infer the cell type composition of a human heart slice (FH6_1000L2_CN74_D1) at the 6.5 PCM stage, using a matched scRNA-seq data as the reference. Since the data lacks single-cell resolution, we utilized high-resolution data acquired through in situ sequencing (ISS) technology [34] from the same study for assessment (Fig 4A). The results from AddaGCN exhibited a high degree of consistency between the predicted cell type distributions with the ISS data (Fig 4B). Specifically, AddaGCN accurately identified the spatial localization patterns of various cell types within the cardiac tissue (Fig 4C): atrial cardiomyocytes were predominantly situated within the atrium; fibroblast-like-1 cells were mainly located at the base of the outflow tract (OFT) and in the valve apparatus; and smooth muscle cells were primarily localized in the aorta and pulmonary artery area. These findings are consistent with established medical knowledge [34]. In contrast, DSTG, SD2, SpatialDecon and SPOTlight failed to identify the aforementioned key cell types (Figs 4B and K in S1 Appendix). Since DOT did not yield valid results, we excluded it from subsequent comparative analyses. A previous review study [35] indicated that Myoz2-expressing cardiomyocytes are difficult to identify because many deconvolution methods struggle to distinguish them from ventricular cardiomyocytes. In our analysis, we also observed that although CellDART, STdGCN and Spoint could identify ventricular cardiomyocytes, they were unable to recognize Myoz2-expressing cardiomyocytes (Fig K in S1 Appendix). However, AddaGCN, Tangram and Cell2location accurately identified Myoz2-expressing cardiomyocytes, highlighting their superior performance compared to other methods (Figs 4C and K in S1 Appendix).

Subsequently, we employed AddaGCN to conduct deconvolution on multiple slices across three developmental stages. These stages corresponded to the 4.5-5, 6.5, and 9 PCW heart tissues, with four, nine, and six tissue sections along the dorsal-ventral axis, respectively. The deconvolution results revealed spatiotemporal changes in cell-type composition during human heart development (Fig L in S1 Appendix). These results demonstrated the spatiotemporal formation of fibroblasts as well as the valvular and muscular structures across the three developmental stages (Fig 4D). Notably, ventricular and atrial cardiomyocytes were identified at all three developmental stages, and their spatial localization was highly consistent with the expressions of their corresponding marker genes (Figs 4E and M–O in S1 Appendix). Specifically, smooth muscle cells were present at low abundance and exhibited a diffuse spatial distribution at the earliest stage (PCW 4.5-5), consistent with an immature state lacking organized vascular structure. At PCW 6.5, discrete regions of smooth muscle cells enrichment emerged, suggesting the formation of spatially restricted niches corresponding to nascent vascular compartments. At PCW 9, smooth muscle cells adopted highly localized and contiguous spatial patterns, forming sharply defined clusters indicative of stabilized vascular architecture. In parallel, atrial cardiomyocytes displayed a distinct yet complementary trajectory. While atrial cardiomyocytes were already detectable at early stages, their spatial distribution became progressively more confined and structured over time, ultimately forming coherent domains consistent with atrial compartmentalization at later stages. These spatial patterns are consistent with the established roles of smooth muscle cells in vascular wall formation and atrial cardiomyocytes in chamber specification and functional maturation [34].

### Human breast cancer data

To evaluate the generalizability of AddaGCN, we applied it to analyze the human breast cancer data [30]. We obtained spatial transcriptomics profiles from an estrogen receptor-positive (ER+) sample (CID4535) and a triple-negative breast cancer (TNBC) sample (CID4465). The corresponding scRNA-seq datasets, which are matched with the ST data, serve as the reference for inferring cell-type proportions in the spatially resolved datasets.

**Fig 5:**
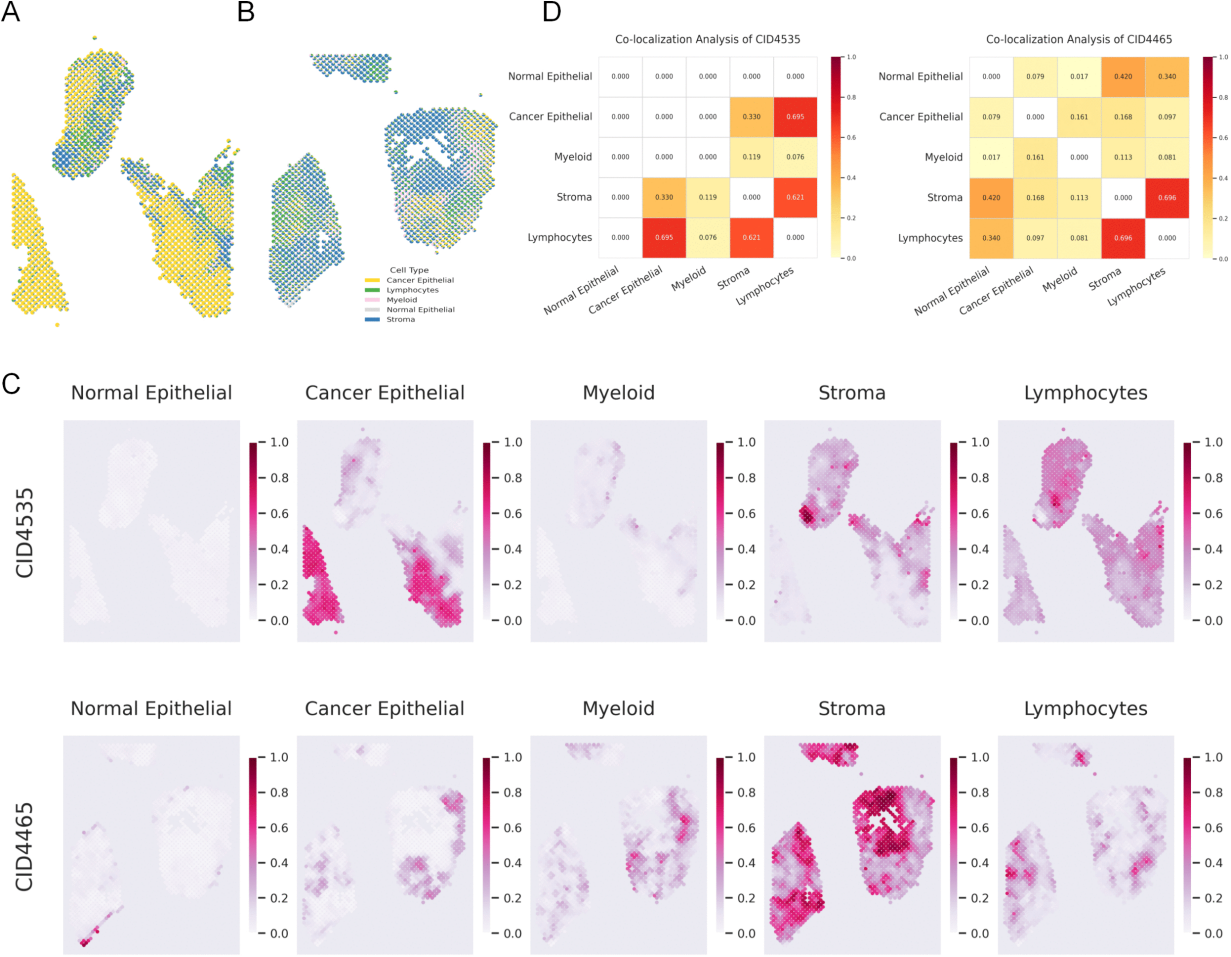
AddaGCN application to human breast cancer. **(A-B)** The pie plot displays the predicted cell-type proportions of AddaGCN in each spot of CID4535 (A) and CID4465 **(B)**. **(C)** The scatter plot displays the predicted proportions of five major celltypes from CID4535 and CID4465. **(D)** The heatmap displays the co-localization score for normal cells, cancer cells, lymphocytes (integration of B-cells, T-cells, and plasmablasts), stroma (integration of cancer-associated fibroblasts, perivascular-like and endothelial cells), and myeloid.

Analysis of the cellular composition of CID4535 (ER+) using AddaGCN showed that cancer epithelial cells were the predominant cell type, followed by stromal cells as the second-most abundant population (Fig 5A). In CID4465 (TNBC), stromal cells were identified as the most prevalent cell type, with cancer epithelial cells being the second-largest population (Fig 5B). Notably, AddaGCN demonstrated high efficiency in reconstructing the spatial distribution of stromal cells in CID4535, indicated by the high agreement between the generated cell-type maps and the ground truth (Figs 5C and P in S1 Appendix). By using AddaGCN, we were able to quantitatively analyze the tumor microenvironment (TME) and visualize these tissues at high resolution. We calculated cell-type co-localization scores (Text B in S1 Appendix) based on the cellular deconvolution results obtained from AddaGCN. The co-localization scores of CID4535 and CID4465 showed substantial differences (Fig 5D), indicating distinct TME features. Furthermore, we were able to analyze intercellular interactions in spatial location. For example, lymphocytes are immune cells that play a crucial role in the humoral immune response, being responsible for antibody production and secretion [36,37]. Accumulating evidence has established tumor-infiltrating lymphocytes (TILs) as clinically significant prognostic biomarkers, with luminal breast cancer subtypes exhibiting improved clinical outcomes associated with elevated TIL infiltration levels [38]. The co-localization heatmap revealed a higher abundance of TILs in CID4535 (ER+) than CID4465 (TNBC), suggesting a more favorable prognosis for CID4535 (ER+) (Fig 5D).

## Discussion

AddaGCN is a graph-based adversarial domain adaptation framework that leverages single-cell RNA-seq data to deconvolve cell-type mixtures in ST, facilitating accurate estimation of spatial cell-type distributions. Compared with existing methods, AddaGCN demonstrated superior performance on various ST datasets. Specifically, AddaGCN successfully reconstructed the mouse brain cortex hierarchies, distinguished tumor sub-regions in pancreatic cancer, and mapped spatiotemporal dynamics in human embryonic heart development, showing strong concordance with histological and ISS data. AddaGCN serves as a powerful tool for spatial tissue analysis, offering clinically relevant insights into cellular organization and holds potential for diagnostic applications.

In practice, we select scRNA-seq references from the same tissue and similar biological contexts, such that major cell types are largely shared between the reference and the ST dataset. For potential novel or unrepresented cell types in spatial data, the model may partially assign their signals to the most similar known cell types. This is a common limitation shared by many reference-based deconvolution approaches. As scRNA-seq resources continue to expand, integrating multiple datasets can further improve reference completeness.

Furthermore, although adversarial domain adaptation does not provide a formal guarantee of preserving class-conditional structure, our framework alleviates this limitation by combining graph-based information propagation with cell-type proportion supervision from pseudo-spots. The resulting latent representations retain biologically meaningful organization, as supported by clustering and visualization analyses, while achieving improved alignment between pseudo- and real-spot domains (Figs Q and R in S1 Appendix). Nevertheless, developing methods with stronger theoretical guarantees for preserving cell-type geometry during domain adaptation remains an important direction for future research.

## Materials and methods

### Data pre-processing

First, the count matrix obtained from the scRNA-seq dataset is normalized to ensure the comparability of gene expression across different cells. After normalization, a log transformation is applied. Specifically, the log-normalization is performed as follows: ${\tilde{X}}_{ij}=\log\left(1+M\frac{X_{ij}}{\sum_{j^{\prime }=1}^{m}X_{ij^{\prime }}}\right)$, where $𝐗\in {\mathbb{R}}^{n\times m^{\prime }}$ is the raw count matrix with *n* cells and $m^{\prime }$ genes, *M* is the scaling factor which is set to 10000. Using the log-normalized expression data, we extract the top 20 highly expressed marker genes for each cell type in the samples through the Wilcoxon rank-sum test. Multiple comparisons are adjusted using the Benjamini-Hochberg method, and the genes are ranked according to their adjusted p-values. All identified cell-type markers are aggregated to form a comprehensive list of marker genes. Subsequently, we selected the intersection between marker genes and detection genes in the ST data and the intersection between marker genes and detection genes in the scRNA-seq dataset for further analysis. Finally, for the scRNA-seq reference dataset, the raw expression data is denoted as ${𝐗}_{ref}\in {\mathbb{R}}^{n_{sc}\times m}$, where $n_{sc}$ is the number of cells and *m* is the number of selected genes. For the ST data, we conduct log-normalization and denote the normalized expression data as ${𝐗}_{r}\in {\mathbb{R}}^{n_{r}\times m}$, where $n_{r}$ is the number of spots and *m* is the number of selected genes.

### Pseudo-ST data generation

The generation of pseudo-ST data involves randomly selecting cell mixtures from scRNA-seq data of the same tissue. Based on our findings, the heterogeneity of pseudo-ST data is a key factor in constructing an effective GCN link graph construction. Traditional pseudo-spot generation methods may lead to an imbalanced pool of pseudo-ST data with limited heterogeneity, particularly due to the imbalanced cell type proportions in the reference single-cell data. To address this issue, we designed a pseudo-ST data generation approach comprising two parts, cell-type-based sampling and cell-based sampling, to incorporate both cell type constraints and randomness. We assume that a total of pseudo-spots ($n_{p}=n_{p_{1}}+n_{p_{2}}$) will be generated, with cell-type-based sampling accounting for 60% to 90% of the total.

Cell-type-based sampling. For each pseudo-spot, we assume $n_{ct}$ contains either 1 or 2 cell types and consider all possible combinations (For example, in the case of three types, the total number of possible combinations is $C_{3}^{1}+C_{3}^{2}=3+3=6$, where $C_{3}^{1}=3$ accounts for one-type combinations and $C_{3}^{2}=3$ accounts for two-type combinations). In each case, we randomly set the proportions of the cell types and then sample cells from the single-cell data accordingly. We calculate the mean expression of the selected cells to obtain the expression for that pseudo-spot. A total of $n_{p_{1}}$ samples are taken from this section.

Cell-based sampling. For each pseudo-spot, we randomly select $n_{c}$ cells from the single-cell reference data. Each selected cell is assigned a random weight to simulate the presence of only a subset of cells within the spatial spot. The weighted average of the gene expression values is assigned to the pseudo-spot. A total of $n_{p_{2}}$ samples are taken from this section.

We suggest that the number of pseudo-spots ($n_{p}$) be set to 2–10 times the number of spots in the real ST data ($n_{r}$). For small datasets, we recommend using a larger multiplier (e.g., 10 times $n_{r}$), while for large datasets, a slightly smaller multiplier may be used. This strategy provides sufficient sampling of spatial cell-type distributions while avoiding unnecessary computational overhead caused by excessive redundancy. The results for sensitivity analysis of the hyperparameters are given in Text C, Figs S and T in S1 Appendix.

After obtaining the pseudo-ST data (Text D in S1 Appendix), we preprocess it using log-normalization and denote the normalized data as ${𝐗}_{p}\in {\mathbb{R}}^{n_{p}\times m}$. The corresponding cell type proportion is denoted as $𝐘\in {\mathbb{R}}^{n_{p}\times K}$, where $y_{i,k}$ is the proportion of *k* -th cell type in the pseudo-spot *i*, *k* is the index of the cell type.

### Link graph construction

In the AddaGCN model, a link graph is constructed for real and pseudo-spots. Let *G* = (*V*, *E*) be an undirected graph, where *V* represents the set of all spots, including both real and pseudo-spots, and the edges *E* are formed based on two aspects: the mutual nearest neighbors (MNN) and the spatial neighbor network (SNN). The MNN graph is constructed according to the similarity of gene expression patterns, while the SNN graph is built based on the spatial locations of real-spots to incorporate spatial information. Specifically, the MNN graph is constructed as follows: KNNs (default value is 30) are detected in a shared embedding space, and only the top 5 MNN pairs are retained; the candidate MNN edges are further validated based on their KNNs (default value is 100) based on top-loading genes. Details and the robustness of graph construction parameters are discussed in Text C and Fig U in S1 Appendix. Edges exist among pseudo-spots, among real-spots, and between pseudo and real-spots.

Edges among pseudo-spots. We construct MNN graph within the pseudo-ST data. The link graph is represented by adjacency matrix ${𝐀}^{\left(1\right)}\in {\mathbb{R}}^{n_{p}\times n_{p}}$, where $A_{ij}^{\left(1\right)}=1$ indicates an edge between spot *i* and spot *j*.

Edges among real-spots. The MNN graph among real-spots is constructed and represented by adjacency matrix ${𝐀}^{MNN}\in {\mathbb{R}}^{n_{r}\times n_{r}}$.

To incorporate spatial information of real-ST data, we construct SNNs based on spatial coordinates of spots. In the SNN graph, an edge exists between spot *i* and spot *j* if their Euclidean distance is less than a predefined radius *r*. Specifically, for 10x Visium data, we configure the graph to include the six nearest neighbors for each spot. For other datasets, *r* is chosen empirically to ensure that each spot has an average of 6–15 neighbors. The SNN graph is represented by adjacency matrix ${𝐀}^{SNN}\in {\mathbb{R}}^{n_{r}\times n_{r}}$, where the entries are 1 if the spots are connected. The two adjacency matrices are merged into ${𝐀}^{\left(2\right)}\in {\mathbb{R}}^{n_{r}\times n_{r}}$, where $A_{ij}^{\left(2\right)}=1$ if $A_{ij}^{MNN}=1\text{and}A_{ij}^{SNN}=1.$

Edges between pseudo and real-spots. The MNN graph between pseudo and real-spots is constructed and represented by adjacency matrix ${𝐀}^{\left(3\right)}\in {\mathbb{R}}^{n_{p}\times n_{r}}$.

The combined adjacency matrix is formed as


$$\begin{array}{c} 𝐀=(\begin{array}{cc} {𝐀}^{\left(1\right)} & {𝐀}^{\left(3\right)}\\ ({𝐀}^{\left(3\right)}{)}^{T} & {𝐀}^{\left(2\right)} \end{array}). \end{array}$$

and then symmetrically normalized as


$$\begin{array}{c} \tilde{𝐀}={𝐃}^{-1/2}(𝐀+𝐈){𝐃}^{-1/2}, \end{array}$$

where 𝐃 is the diagonal degree matrix of 𝐀+𝐈 and 𝐈 denotes the identity matrix. Ablation studies demonstrate the superior performance of the link graph construction strategy in AddaGCN (Fig V in S1 Appendix).

### GCN-based feature extractor

To learn low-dimensional embeddings of spots from high-dimensional gene expression profiles, we construct a GCN-based feature extractor $f(\cdot ):{\mathbb{R}}^{N\times m}\to {\mathbb{R}}^{N\times 64}$ by incorporating the link graph among pseudo and real-spot. The input of f(·) is the normalized expression matrix $𝐗=[{𝐗}_{p},{𝐗}_{r}]\in {\mathbb{R}}^{N\times m},N=n_{r}+n_{p}$ and the normalized adjacency matrix $\tilde{𝐀}$. In f(·), two GCN layers are used with each layer represented as a nonlinear function:


$$\begin{array}{c} 𝐙=[{𝐙}_{p},{𝐙}_{r}]=f(𝐗,\tilde{𝐀})=\sigma (\tilde{𝐀}\sigma (\tilde{𝐀}{𝐗}^{T}{𝐖}^{\left(0\right)}){𝐖}^{\left(1\right)}), \end{array}$$

where $𝐙\in R^{N\times 64}$ are embedding features, ${𝐖}^{\left(l\right)},l=0,1$ is the weight matrix of the *l* -th layer that needs to be learned during training process, and σ(·) denotes the rectified linear unit (R eLU) activation function.

### Source classifier

The source classifier $S(\cdot ):{\mathbb{R}}^{N\times 64}\to {\mathbb{R}}^{N\times K}$ is used to learn cell-type proportions of each spot. Architecturally, it is formed by a linear transformation layer that operates on the embeddings generated by GCN-based feature extractor and outputs the predicted cell-type proportions. We define the source classification loss on pseudo-spots as:


$$\begin{array}{c} L_{S}=\text{KLD}({𝐘}_{p}\|S(f({𝐗}_{p},\tilde{𝐀})))=-\sum_{i=1}^{n_{p}}\sum_{k=1}^{K}y_{i,k}\ln\left[{\hat{y}}_{i,k}\right], \end{array}$$

where ${\hat{y}}_{i,k}$ is the predicted proportion of *k* -th cell type in pseudo-spot *i* by the source classifier.

### Domain classifier

The domain classifier $D(\cdot ):{\mathbb{R}}^{N\times 64}\to {\mathbb{R}}^{N\times 1}$ is used to distinguish whether the data originates from pseudo (label = 1) or real-spots (label = 0). The D(·) consists of two fully connected layers. The first layer has a 32-dimensional output and is connected to the embeddings from GCN-based feature extractor. After applying the ELU activation function, the second layer is applied to classify whether a spot is a pseudo-spot or a real-spot. We define the domain classifier loss, which measures the probability of correctly assigning a given spot to its real domain label (pseudo or real), as


$$\begin{array}{c} L_{D}=-\frac{1}{N_{p}}\sum_{i=1}^{N_{p}}\log D_{i}\left(f\left(X_{p},\tilde{A}\right)\right)-\frac{1}{N_{r}}\sum_{j=1}^{N_{r}}\log\left(1-D_{j}\left(f\left(X_{r},\tilde{A}\right)\right)\right). \end{array}$$

Using inverted domain labels, the loss is defined as


$$\begin{array}{c} L_{D,I}=-\frac{1}{N_{r}}\sum_{i=1}^{N_{r}}\log D_{i}\left(f\left(X_{r},\tilde{A}\right)\right)-\frac{1}{N_{p}}\sum_{j=1}^{N_{p}}\log\left(1-D_{j}\left(f\left(X_{p},\tilde{A}\right)\right)\right). \end{array}$$

### AddaGCN Algorithm

The model training process involves an initialization phase followed by two optimization steps Text D in S1 Appendix. During initialization, the GCN-based feature extractor f(·) and source classifier S(·) are trained to learn the cell-type proportions of pseudo-spots by minimizing $L_{S}$. In optimization step 1, we fix the weights of the domain classifier D(·) and train the GCN-based feature extractor f(·) and source classifier S(·) by minimizing


$$\begin{array}{c} L_{\text{total}}=L_{S}+\alpha L_{D}, \end{array}$$

where α (with a default value of 0.1) is a hyperparameter that balances the source classification loss and the domain classification loss (Text C and Fig W in S1 Appendix). In optimization step 2, with domain labels inverted, we train the domain classifier D(·) to minimize $L_{D,I}$, while keeping the weights of the GCN feature extractor f(·) and source classifier S(·) fixed. These two optimization steps are iteratively alternated for 1000 cycles, resulting in a trained AddaGCN model capable of predicting cell-type proportions for real-ST data. More details on model training and parameter settings are provided in Text C and Table D in S1 Appendix.

## Supporting information

S1 Appendix Supplementary materials. Text A–D, Tables A–E and Figs A–W.
(PDF)
