# Supplementary material for: AddaGCN: Spatial transcriptomics deconvolution using graph convolutional networks with adversarial discriminative domain adaptation
Source: PLoS Comput Biol. 2026 Aug 5;22(8):e1014609. doi: 10.1371/journal.pcbi.1014609 (PMC13466050; doi:10.1371/journal.pcbi.1014609)
Supplement: Supplementary file 1 [file pcbi.1014609.s001.pdf]

Supplementary Information for

AddaGCN: spatial transcriptomics deconvolution using graph convolutional networks with adversarial discriminative domain adaptation

Shuzhen Ding[1,3], Zhou Yu[1], Jingsi Ming[1,2*]

[1] *KLATASDS-MOE, School of Statistics, East China Normal University, Shanghai, China*

[2] *Academy of Statistics and Interdisciplinary Sciences, East China Normal University, Shanghai, China*

[3] *College of Mathematics and Systems Sciences, Xinjiang University, Urumqi, China*

[*] Corresponding author. Email: jsming@fem.ecnu.edu.cn

Text A

The seqFISH+ dataset

We analyzed a publicly available seqFISH+ dataset [1], which encompasses 523 cells with corresponding cell type annotations and spatial coordinates from the mouse cortex, all at single-cell resolution. To generate low-resolution ST data, we aggregated the cells on uniform grids to create simulated spots, with the center of each square serving as the new coordinate. Each simulated spot was designed with a side length of 51.5 μm, approximating low-resolution spots that contain approximately 7.3 cells. Given that cell type labels are known at single-cell level, the ground-truth cell type proportions within these simulated spots could be used for performance evaluation. For deconvolution reference, we used a publicly available scRNA-seq dataset [2], which includes 1,691 cells and identifies six major cell types: astrocytes, endothelial-mural cells (endo_mural), excitatory neurons (eNeuron), inhibitory neurons (iNeuron), microglia cells, and oligodendrocytes (Olig).

The Xenium dataset

Another dataset has been obtained from the Xenium In Situ platform [3], which contains 159,226 cells and 313 genes with corresponding nineteen cell type annotations and spatial coordinates from a human breast carcinoma sample, all at single-cell resolution. To generate low-resolution ST data, we select a small piece from the slice containing 14,867 cells. we aggregated the cells on uniform grids to create simulated spots, with the center of each square serving as the new coordinate. Each simulated spot was approximating low-resolution spots that contain approximately 5 cells. Given that cell type labels are known at single-cell level, the ground-truth cell type proportions within these simulated spots could be used for performance evaluation. For deconvolution reference, we used another part that includes cells as single-cell reference data,

which includes 31,693 cells and identifies nineteen major cell types.

The independent breast cancer scRNA-seq reference dataset

To assess the batch-correction capability under realistic cross-study conditions, we performed an additional benchmark experiment using an independent breast cancer scRNA-seq reference dataset (GSE176078) [4], which is completely distinct from the Xenium spatial transcriptomics dataset in terms of study origin, experimental protocol, and sequencing platform. This dataset contains scRNA-seq profiles from 26 primary human breast tumors spanning multiple breast cancer subtypes. For evaluation, we harmonized the annotation systems between the Xenium data and the external scRNA-seq reference by merging biologically similar subtypes and focused on eight common cell types (Endothelial, Myeloid, T-cells, B-cells, Normal Epithelial, CAFs, PVL and Cancer Epithelial). We further randomly downsampled 30,000 cells from the external scRNA-seq dataset as the final reference for computational efficiency.

The PDAC data

We established the ground truth boundaries of cancer regions for slice PDAC-A through manual annotations of H&E-stained images (Fig I). The annotation data were obtained from https://github.com/YMa-lab/CARD/tree/master/data and served as the gold standard for calculating spatial matching metrics, including F1-score and false positive rate (FPR).

Text B

To evaluate the effectiveness of cell type decomposition methods, similar to the Benchmarking article [5], we used Pearson correlation coefficient (PCC), structural similarity (SSIM), root mean square error (RMSE), and Jensen-Shannon divergence (JSD) on a simulated dataset to quantify the difference between predicted and actual cell type ratios at each point. On a real dataset, we used PCC to measure the consistency between predicted cell type ratios and marker gene expression. For one spot, higher PCC value, higher SSIM value, lower RMSE value and lower JSD value indicate better prediction accuracy. These four measurement indicators are defined as follows:

PCC (Pearson correlation coefficient) is used to measure the similarity between predicted cell-type proportions and real cell-type proportions within each spot. It can be expressed as:

$$PCC = \frac{E[(P_i - p_i)(\widehat{P}_i - \hat{p}_i)]}{\sigma_i \widehat{\sigma}_i},$$

Where P_i and $\widehat{P}_i$ are the true and predicted cell type proportion vectors of the i-th spot, respectively; p_i and $\hat{p}_i$ are the average proportion vectors of spot i in the ground truth and the predicted result, respectively; σ_i and $\widehat{\sigma}_i$ are the standard deviations of the true and predicted cell type distribution in spot i, respectively.

SSIM (Structural Similarity) is a metric for image similarity, essentially comparing two matrices. Thus it can also be used to quantify the effectiveness of deconvolution. The calculation is performed as follows:

$$SSIM = \frac{(2p_i\hat{p}_i + C_1^2)(2cov(P_i, \widehat{P}_i))}{(p_i^2 + \hat{p}_i^2 + C_1^2)(\sigma_i^2 + \widehat{\sigma}_i^2 + C_2^2)},$$

where C_1 and C_2 are 0.01 and 0.03 respectively; $cov(P_i, \widehat{P}_i)$ is the the covariance between the cell type proportion vectors in the ground truth (P_i) and the predicted result ($\widehat{P}_i$); p_i, $\widehat{p}_i$, σ_i and $\widehat{\sigma}_i$ are similar to those for calculating the PCC value.

RMSE (Root Mean Square Error) is employed to quantify the dispersion between the predicted and real cell-type proportions within each spot. The calculation is detailed below:

$$RMSE = \sqrt{\frac{1}{K}\sum_{k=1}^{K}(z_{ik} - \hat{z}_{ik})^2},$$

where z_{ik} and $\hat{z}_{ik}$ are the z-scores of P_{ik} and $\widehat{P}_{ik}$.

JSD (Jensen-Shannon Divergence) uses Kullback-Leibler divergence to measure the difference between two distributions of predicted cell-type proportions and real cell-type proportions. The JSD is formulated as follows:

$$JSD = \frac{1}{2}KL\left(P_i \middle| \frac{P_i+\widehat{P}_i}{2}\right) + \frac{1}{2}KL\left(\widehat{P}_i \middle| \frac{P_i+\widehat{P}_i}{2}\right),$$

$$KL(f|g) = \sum_{i=0}^{K}(f_i \times log\frac{f_i}{g_i}),$$

where $KL(f|g)$ denotes the Kullback–Leibler (KL) divergence between two distributions f and g.

Cell type co-localization score. Cell–cell interactions are primarily mediated by specific receptor–ligand binding events. Consequently, the spatial co-localization of distinct cell types can serve as a proxy for identifying potential intercellular communication networks within tissues. To systematically evaluate the co-localization patterns between two cell populations in a ST data, we implemented a quantitative metric termed the cell type co-localization score, defined as $S(A, B)$. $S(A, B)$ can be calculated using the following functions:

$$S(A, B) = \frac{\sum_i S_{i,A}=1 \text{ and } S_{i,B}=1}{\sum_i S_{i,A}=1 \text{ or } S_{i,B}=1}.$$

The co-localization score $S_{i,A} = 1$ indicates the presence of cell type A at a given location i, with values ranging from 0 (no co-localization) to 1 (strong co-localization). Since AddaGCN predicts all cell type proportions to be greater than 0, we applied a 0.15 threshold to the predicted proportion of cell type A to ensure meaningful analysis. When the predicted proportion of cell type A within a spot is below the threshold, we set $S_{i,A} = 0$. This threshold was selected based on the specific characteristics of our 10X Visium human breast cancer dataset.

Text C

Hyperparameter Sensitivity Analysis

We suggest that the number of pseudo-spots (n_p) be set to 2-10 times the number of spots in the real ST data (n_r). For the seqFISH+ data where n_r=71, AddaGCN achieves satisfactory performance when n_p=700 (approximately 10 times n_r) (Fig S). For the Xenium data where n_r=4,237, AddaGCN achieves consistently robust performance when n_p ranges from 5,000 to 30,000 (approximately 2-10 times n_r) (Fig T). As a result, for small datasets, we recommend using a larger multiplier (e.g., 10 times n_r), while for large datasets, a slightly smaller multiplier may be used. This strategy provides sufficient sampling of spatial cell-type distributions while avoiding unnecessary computational overhead caused by excessive redundancy.

For the parameter the number of marker genes selected per cell type. Our analysis indicates that a small number of markers (20 genes) is sufficient to achieve stable deconvolution performance (Figs S and T). Increasing the marker set beyond this range does not yield substantial improvements and may introduce noise from less informative genes, as well as increased computational cost.

For the parameter n_{ct} (the number of fixed cell types per pseudo-spot), our analysis indicates that small values (n_{ct} = 1 or 2) are sufficient to capture local cell-type composition patterns (Figs S and T). Increasing n_{ct} beyond this range leads to performance degradation, likely due to the introduction of irrelevant cell-type mixtures and increased noise, as well as higher computational cost.

For the optimal proportion of cell type-based sampling in the total number of pseudo-spots, ranging the ratio from 0% to 90%. Our analysis indicates that a moderate proportion (60% to 90%) is sufficient to achieve stable model performance (Figs S and T). Deviating from this range, particularly at lower ratios, leads to performance degradation, likely due to insufficient preservation of cell-type-specific signals and increased imbalance in pseudo-spot composition.

For the key parameters in graph construction, the model performance remains relatively stable across a broad range of parameter values. In particular, comparable performance is observed when k_1 is between 20-40, k_2 between 2-8, and k_3 between 50-200. These findings indicate that AddaGCN is not overly sensitive to specific graph construction parameters, and that the default settings fall within a robust operating range. This robustness ensures that reliable performance can be achieved without extensive parameter tuning.

For parameter α which controls the balance between the cell-type classification objective and the domain adaptation objective, AddaGCN achieves stable and consistently strong performance when α is set in the range of 0.05-0.1. When α is too small, domain alignment becomes insufficient, whereas excessively large values may overemphasize domain invariance at the expense of cell-type discrimination. Based on these observations, we selected α = 0.1 as a default setting for all datasets, as it provides a good balance between accuracy and robustness.

Experimental setup

In all parameter settings of our model, during the generation of pseudo-ST data, the number of cells n_c for each pseudo-spot depends on the platform resolution (Visium: 8). The total number of pseudo-spots n_p is approximately 2-10 times the number of real-spots, with larger dataset required fewer. The number of marker genes selected in the pre-processing step is set to a default value of 20. In cell-type-based sampling, we assume each pseudo-spot contains either n_{ct} = 1 or 2 cell types and the cell-type-based sampling accounting for 60% to 90% of the total pseudo-spots. The pseudo-ST data is partitioned as follows: 81% is assigned to the training set, 9% to the validation set, and 10% to the test set. The test set serves to evaluate the model's generalization ability on unseen data, while the validation set is used to monitor the model's performance during training, e.g., for early stopping, to prevent overfitting on the training set. For parameter optimization, we employ the Adam optimizer [6] with a learning rate of 0.001.

All baseline methods were implemented following the parameter settings recommended in their original publications or official software packages to ensure a fair comparison. For methods with unspecified or ambiguous parameters, we adopted commonly used or recommended settings from the literature and ensured comparable experimental conditions across methods. For example, in pseudo-spot-based approaches, when the number of pseudo-spots was not explicitly defined, we selected values within a reasonable range consistent with prior studies to maintain comparability across methods. For unspecified parameters in deconvolution methods based on pseudo-ST data (CellDART, STdGCN, DSTG, SD2 and Spoint), we set them to be the same with those of AddaGCN. The key parameter settings are presented in Table D.

Text D

Algorithm 1： Pseudo-ST data generation

Input: Hyper-parameters n_c, n_{p_1}, n_{p_2}, $n_p = n_{p_1} + n_{p_2}$, one hot label matrix C of cell type, the raw scRNA-seq reference data X_{ref}

Output: $X_p \in \mathbb{R}^{n_p \times m}$, $Y \in \mathbb{R}^{n_p \times K}$

Initialize: $X_{p1} \leftarrow 0_{n_{p_1} \times m}$; $Y_{p1} \leftarrow 0_{n_{p_1} \times K}$; $X_{p2} \leftarrow 0_{n_{p_2} \times m}$; $Y_{p2} \leftarrow 0_{n_{p_2} \times K}$

Cell-type-based sampling:

for s in $1{:}\, n_{p_1}$ **do**

For pseudo spot s, fix the number of cell types in each pseudo-spot (n_{ct}=1 or 2) and sample n_c cells from the single-cell data X_{ref} based on the composition. The expression matrix of the selected cells is denoted as $Q \in \mathbb{R}^{n_c \times m}$;

Calculate the expression $X_{p_1}[s,] = \frac{\sum_j Q_j}{n_c}$ and proportion $Y_{p_1}[s,]$ of the pseudo-spot;

end

Cell-based sampling:

for s in $1{:}\, n_{p_2}$ **do**

Random select n_c cells from the single-cell data and denote the set of sample indexes as I_s;

Generate mixed weights and normalize them

$$P_s \leftarrow \frac{U_s}{\|U_s\|_1},\ U_s \sim Uniform([0,1]^{n_c})$$

Calculate the expression and proportions of the pseudo-spot

$$X_{p_2}[s,] \leftarrow \sum_{j=1}^{n_c} P_s \cdot X_{ref}[I_{s,}]$$

$$Y_{p_2}[s,] \leftarrow \sum_{j=1}^{n_c} P_s \cdot C[I_{s,}]$$

end

Merge the data obtained from two sampling methods

$$\widetilde{X}_p = Concatenate(X_{p_1}, X_{p_2})$$

$$Y = Concatenate(Y_{p_1}, Y_{p_2})$$

Apply log-normalization to the pseudo-ST data and obtain X_p.

Algorithm 2: Stochastic gradient descent training of AddaGCN

Input: Hyper-parameters α, combined adjacency matrix $\widetilde{A}$, number of iterations T, $X_r \in \mathbb{R}^{n_r \times m}$, $X_p \in \mathbb{R}^{n_p \times m}$, $Y \in \mathbb{R}^{n_p \times K}$

Output: the GCN-based feature extractor $f(\cdot)$, source classifier $S(\cdot)$ and domain classifier $D(\cdot)$

Initialization:

The GCN-based feature extractor $f(\cdot)$ and source classifier $S(\cdot)$ are trained by minimizing

$$L_S = KLD(Y_p || S(f(X_p, \widetilde{A}))) = -\sum_{i=1}^{n_p} \sum_{k=1}^{K} y_{i,k} \ln [\hat{y}_{i,k}].$$

for t in $1{:}T$ **do**

Optimization step 1:

Fix the weights of the domain classifier $D(\cdot)$ and train the GCN-based feature extractor $f(\cdot)$ and source classifier $S(\cdot)$ by minimizing

$$L_{total} = L_S + \alpha L_D,$$

$$L_D = -\log D\left(f\left(X_p, \widetilde{A}\right)\right) - \log\left(\left[1 - D\left(f\left(X_r, \widetilde{A}\right)\right)\right]\right).$$

Optimization step 2:

Fix the weights of the GCN feature extractor $f(\cdot)$ and source classifier $S(\cdot)$, invert domain labels, and train the domain classifier $D(\cdot)$ by minimizing

$$L_{D,I} = -\log D\left(f\left(X_r, \widetilde{A}\right)\right) - \log\left(\left[1 - D\left(f\left(X_p, \widetilde{A}\right)\right)\right]\right).$$

end

Supplementary Tables

Table A. Cell type compositions in five scenarios and the ST data (%)

Cell type	Scenario 1	Scenario 2	Scenario 3	Scenario 4	Scenario 5	ST
Invasive_Tumor	31.57	27.64	1.35	11.77	9.80	32.49
Stromal	18.36	29.72	33.45	34.74	36.91	10.63
DCIS_2	11.10	0.71	5.46	2.82	6.92	25.46
Macrophages_1	7.37	5.87	6.59	7.13	6.84	6.81
CD4+_T_Cells	6.40	4.81	4.99	4.99	5.78	3.73
Endothelial	5.14	7.85	4.03	5.52	6.69	4.87
CD8+_T_Cells	4.72	3.58	4.82	4.52	4.88	2.91
Myoepi_ACTA2+	3.41	2.13	7.34	4.66	4.63	5.78
Prolif_Invasive_Tumor	3.30	2.24	0.09	1.57	1.09	2.75
DCIS_1	2.74	7.86	19.85	12.01	8.65	0.36
B_Cells	2.71	2.69	4.33	3.93	3.59	2.10
Macrophages_2	0.75	1.32	0.87	1.53	1.59	0.59
Myoepi_KRT15+	0.66	1.19	4.92	2.95	1.01	0.07
Perivascular-Like	0.44	1.10	0.65	0.37	0.44	0.23
T_Cell_&_Tumor_Hybrid	0.44	0.41	0.08	0.18	0.15	0.69
Stromal_&_T_Cell_Hybrid	0.35	0.25	0.51	0.49	0.30	0.22
IRF7+_DCs	0.26	0.33	0.37	0.44	0.46	0.07
LAMP3+_DCs	0.19	0.22	0.20	0.22	0.16	0.13
Mast_Cells	0.08	0.08	0.11	0.16	0.12	0.09

Table B. Runtime and memory usage of different methods

Method	Xenium (4,237 spots)		Xenium (18,179 spots)	
	Time (s)	Memory (MB)	Time (s)	Memory (MB)
AddaGCN	539.09	2219.14	2522.35	3678.99
CellDART	98.38	2637.05	612.16	3176.66
STdGCN	41.77	1902.38	CUDA out of memory	
Spoint	68801.12	2324.01	71951.47	2798.02
Tangram	6.87	1272.12	27.81	1480.91
SpatialDecon	40.74	580.17	184.21	734.46
Cell2location	430.01	3329.24	613.67	8249.05
DOT	39.03	109.19	160.82	354.49
SPOTlight	14672.88	8425.57	kernel died	

Table C. Performance comparison of cancer region identification across different methods

Methods	Precision	Recall	F1-score	FPR
AddaGCN	0.86	0.84	0.85	0.07
CellDART	0.85	0.12	0.22	0.01
STdGCN	0.85	0.5	0.63	0.04
DSTG	0	0	0	0
SD2	0	0	0	0
Spoint	0.77	0.88	0.82	0.13
Tangram	0.88	0.44	0.59	0.03
SpatialDecon	0.94	0.54	0.68	0.02
Cell2location	0.89	0.82	0.85	0.05
DOT	0.81	0.73	0.77	0.08
SPOTlight	0	0	0	0.02

Table D. Hyperparameter settings for simulated and real datasets

Methods	The number of pseudo spot (n_p)	The number of cells in each spot (n_c)	Cell type number constraint (n_{ct})	Marker genes
seqFISH+				
CellDART	700 (5~10 times)	7	None	100
STdGCN	30000	8-12	Yes/4	100
DSTG	700	5~9	None	2000 genes
SD2	700	5~9	None	2000 dropout genes
AddaGCN	700	7	Yes/1 (70.3%)	20
Spoint	10000	Normal distribution	Normal distribution	200
Xenium				
CellDART	10000	5	None	100
STdGCN	10000	2-6	Yes/4	100
DSTG	10000	2-6	None	2000 genes
SD2	10000	2-6	None	2000 dropout genes
AddaGCN	10000	5	Yes/1 (60.4%)	20
Spoint	10000	Normal distribution	Normal distribution	200
PDAC				
CellDART	3000	8	None	100
STdGCN	30000	8-12	Yes/4	100
DSTG	1500	2~8	None	2000 genes
SD2	1500	10-20	None	2000 dropout genes
AddaGCN	1500	8	Yes/2 (69.3%)	20
Spoint	10000	Normal distribution	Normal distribution	200
Development Human Heart				
CellDART	2500	8	None	100
STdGCN	30000	8-12	Yes/4	100
DSTG	1000	2~8	None	2000 genes
SD2	1000	10-20	None	2000 dropout genes
AddaGCN	1000	8	Yes/2(75%)	20
Spoint	10000	Normal distribution	Normal distribution	200
Human Breast Cancer				
CellDART	5000	8	None	100
STdGCN	5000	8-12	Yes/4	100
DSTG	5000	2~8	None	2000 genes
SD2	5000	10-20	None	2000 dropout genes
AddaGCN	5000	8	Yes/2(78.3%)	20
Spoint	10000	Normal distribution	Normal distribution	200

Table E. The number of spots and genes for each analyzed datasets

Datasets	Slice Name	Spot Number	Gene Number
seqFISH+	seqFISH+	71	9962
Xenium	Xenium	4237	313
PDAC	PDAC-A	428	15324
	PDAC-B	224	19736
Human breast cancer data	CID4535	1051	15128
	CID4465	1211	15128
The developing human heart data	FH5_1000L3_CN20_C1	55	38936
	FH5_1000L3_CN20_C2	59	38936
	FH5_1000L3_CN20_D1	69	38936
	FH5_1000L3_CN20_D2	55	38936
	FH6_1000L2_CN73_E1	103	38936
	FH6_1000L2_CN73_E2	104	38936
	FH6_1000L2_CN73_D2	159	38936
	FH6_1000L2_CN73_C2	186	38936
	FH6_1000L2_CN74_E1	214	38936
	FH6_1000L2_CN74_E2	209	38936
	FH6_1000L2_CN74_D1	178	38936
	FH6_1000L2_CN74_D2	182	38936
	FH6_1000L2_CN74_C1	180	38936
	FH9_1000L3_CN31_C1	238	38936
	FH9_1000L3_CN31_C2	242	38936
	FH9_1000L3_CN31_D1	243	38936
	FH9_1000L3_CN31_D2	226	38936
	FH9_1000L3_CN31_E1	198	38936
	FH9_1000L3_CN31_E2	211	38936

Supplementary Figures

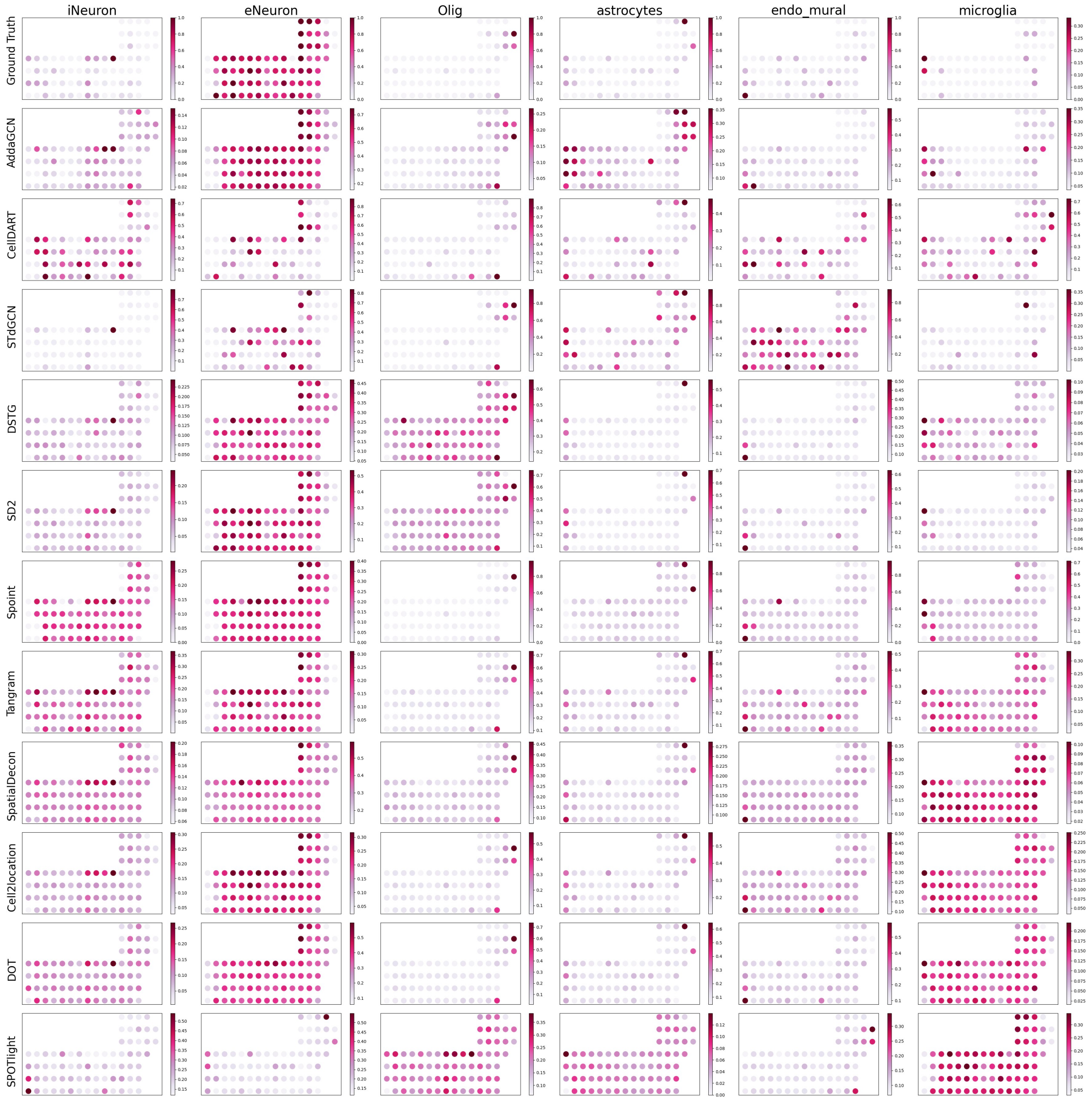

Fig A. The true and inferred proportions of six major cell types at each spatial position using different methods on the seqFISH+ simulated data.

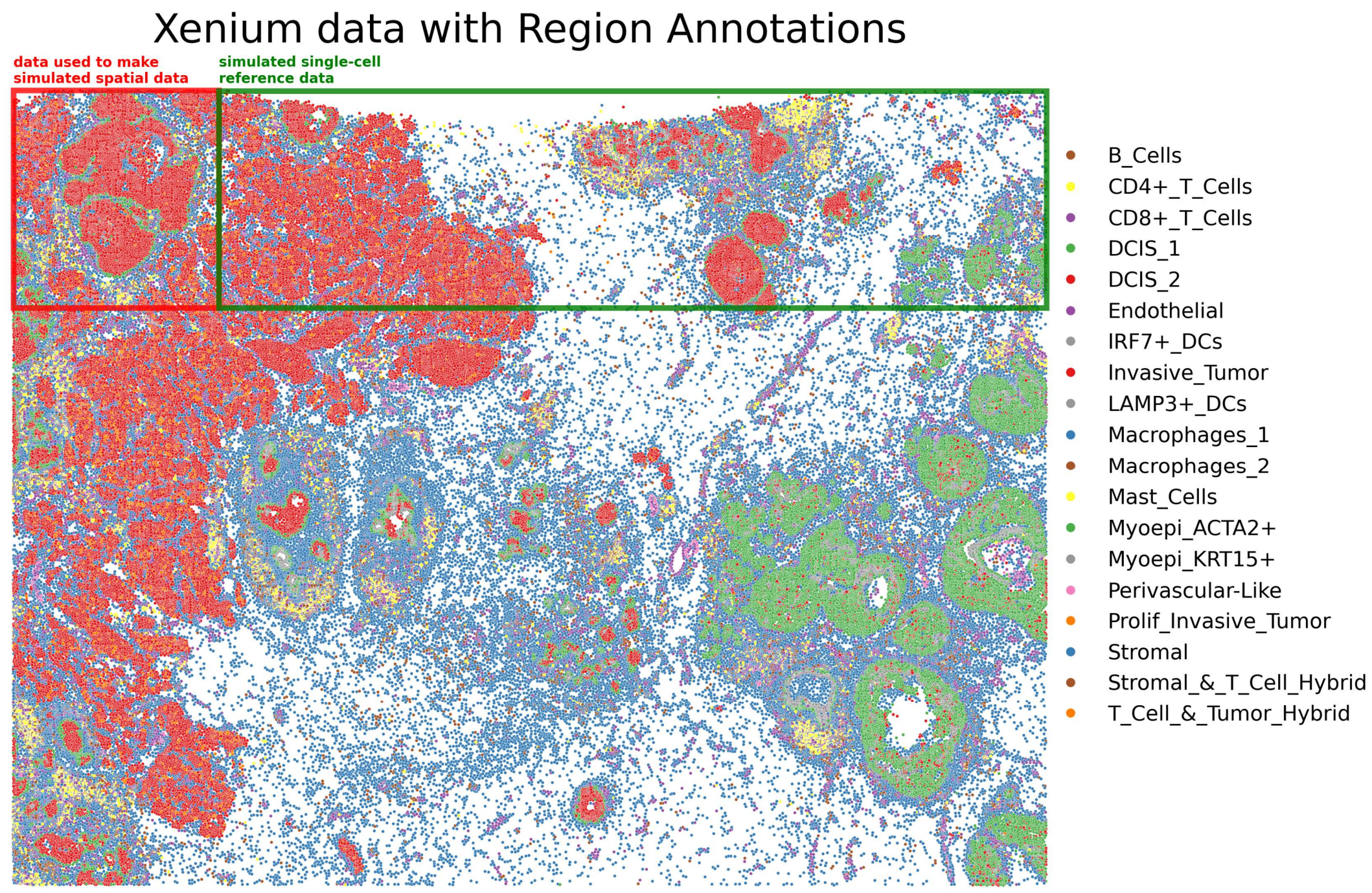

Fig B. The simulated dataset using Xenium dataset. Xenium cells are divided into three subsets. The red box part is used to make pseudo-spots by aggregating the cells within each grid, and the green part is regarded as a paired scRNA-seq reference.

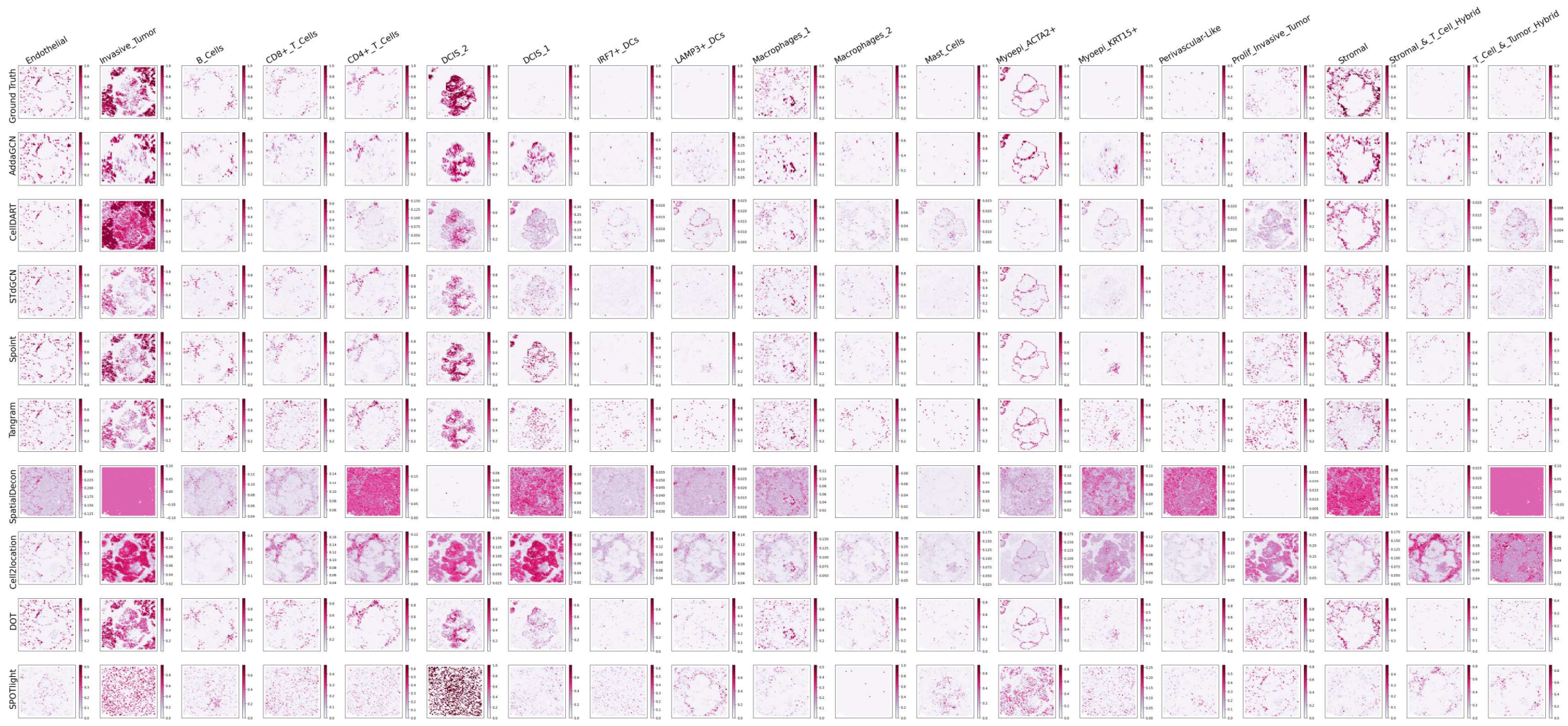

Fig C. The true and inferred proportions of nineteen cell types at each spatial position using different methods on the Xenium simulated data.

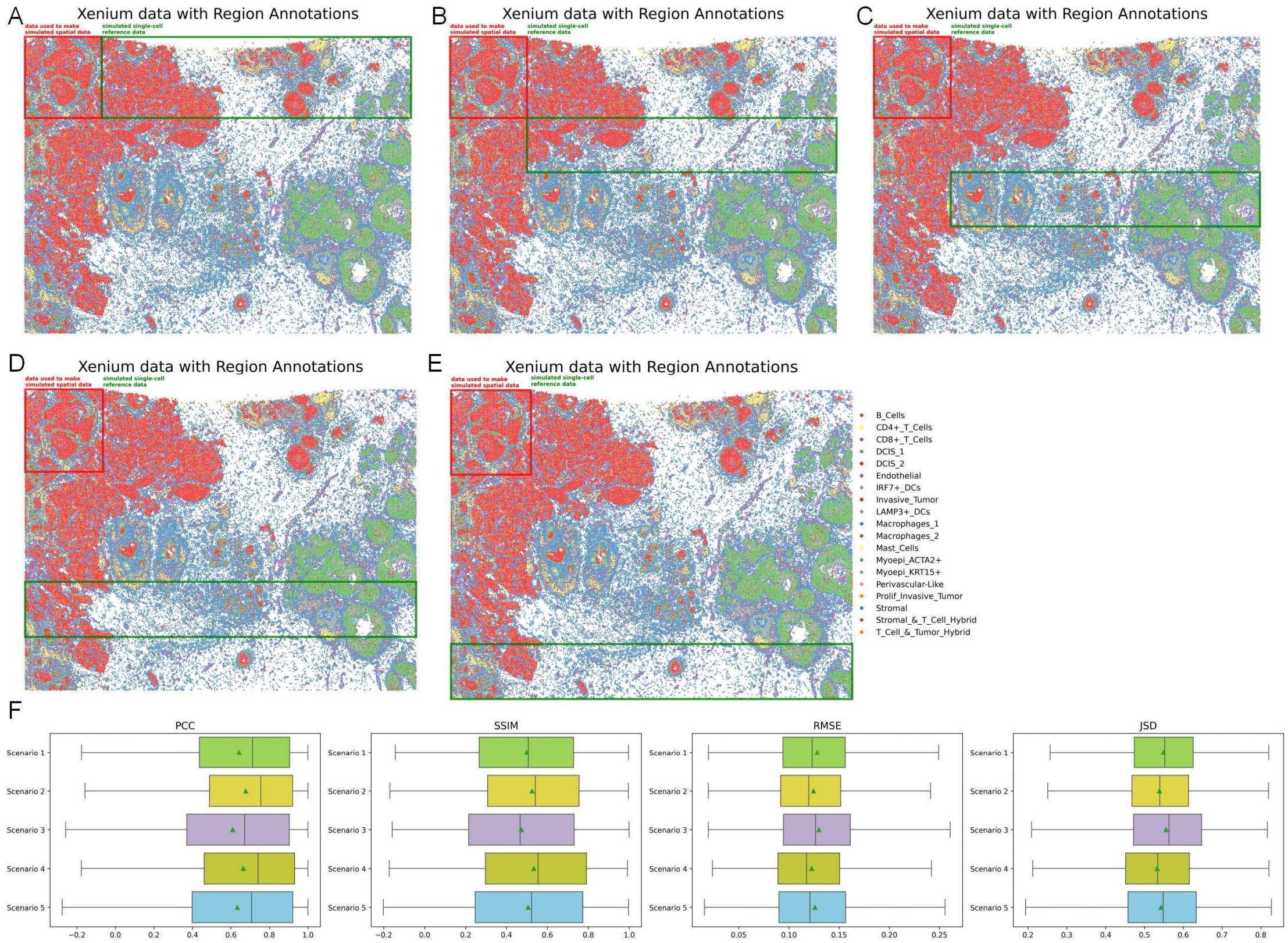

Fig D. The deconvolution performance of AddaGCN on five simulated datasets (A, Scenario 1; B, Scenario 2; C, Scenario 3; D, Scenario 4; E, Scenario 5) using Xenium dataset. The red box part is used to make simulated spots by aggregating the cells with in each grid, and the green box part is regarded as the scRNA-seq reference. (F) Boxplots of PCC, SSIM, RMSE and JSD of each scenario in predicting the cell type distribution of spots.

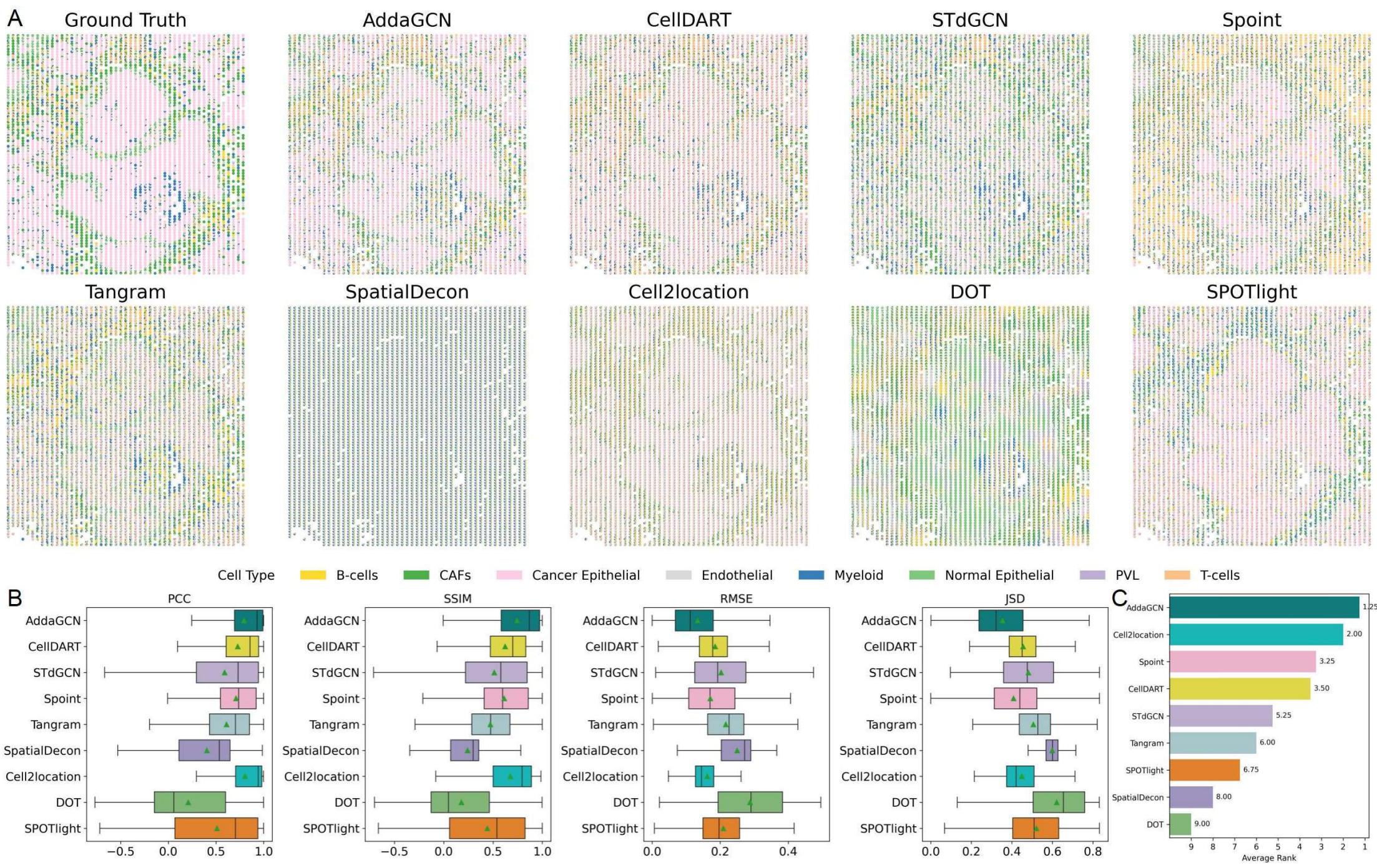

Fig E. Benchmark evaluation on the Xenium data using external single-cell reference. (A) A spatial scatter pie plot displays the true and the inferred cell-type composition of Xenium data from different deconvolution methods. (B)

Boxplots of PCC, SSIM, RMSE and JSD of each deconvolution method in predicting the cell type distribution of spots. (C) Bar plots of average rank (aggregated from PCC, SSIM, RMSE and JSD) of prediction accuracy on the Xenium data.

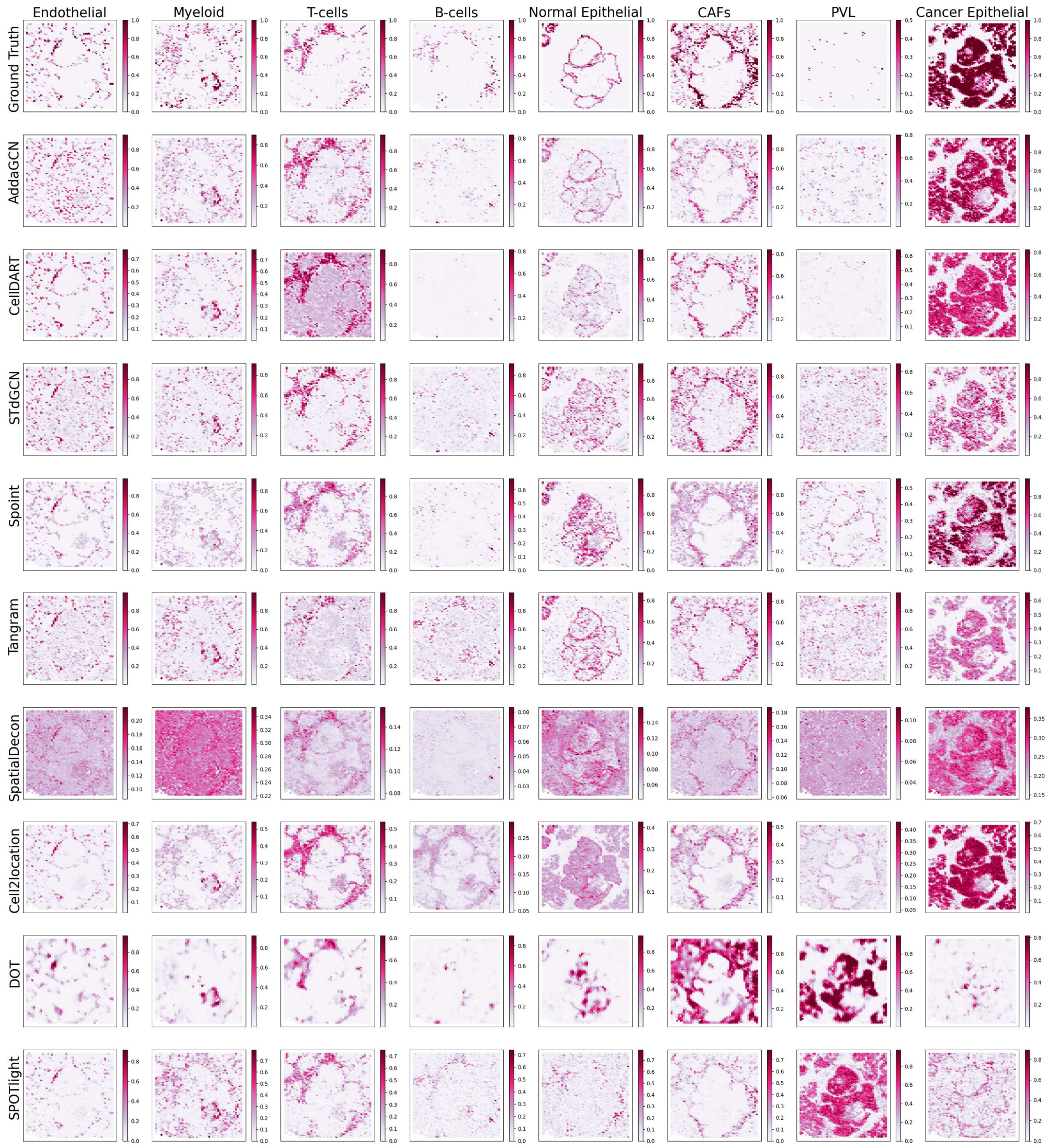

Fig F. The true and inferred cell-type proportions at each spatial position using different methods on the Xenium simulated data using external single-cell reference.

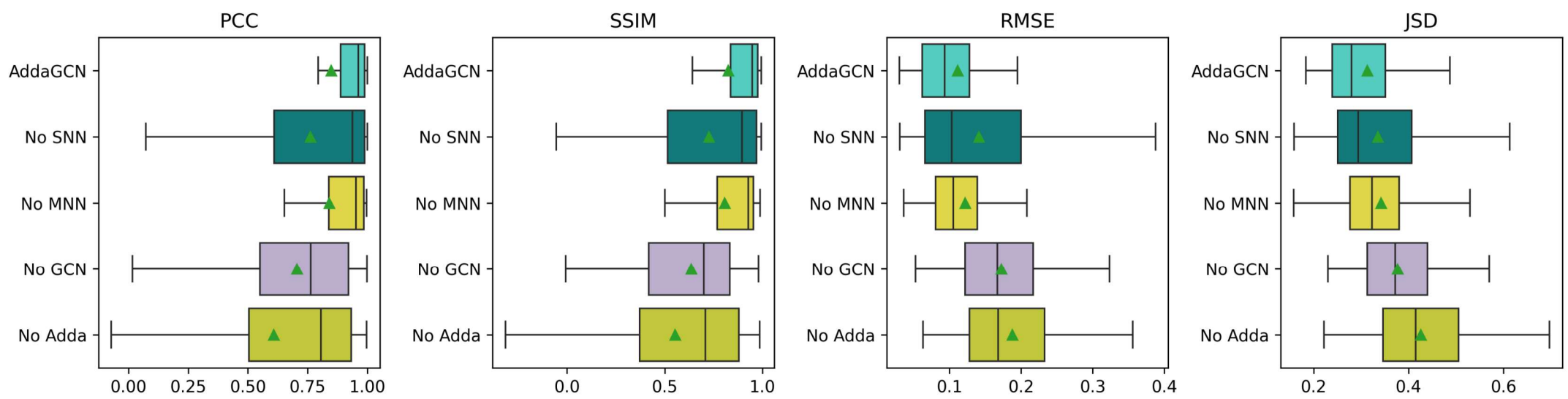

Fig G. The ablation study on the seqFISH+ data comparing the original AddaGCN and the other four conditions: no SNN, no MNN, no GCN and no Adda. Boxplots of PCC, SSIM, RMSE and JSD of the above 5 conditions in predicting the cell type distribution of spots. Higher PCC and SSIM, lower RMSE and JSD indicate better performance.

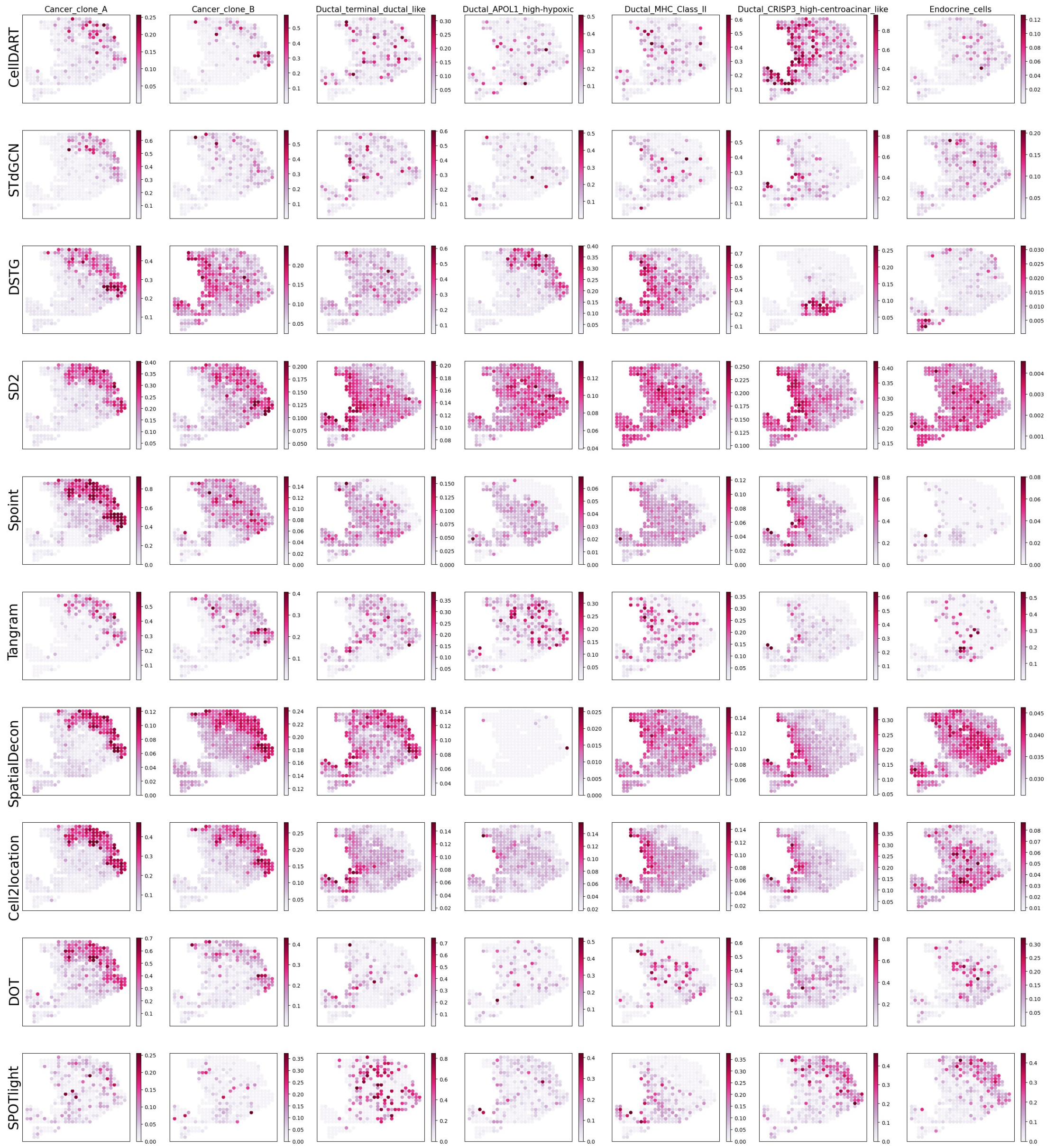

Fig H. The proportion of each of the cell types inferred by different methods is displayed on each spatial location of PDAC-A slice.

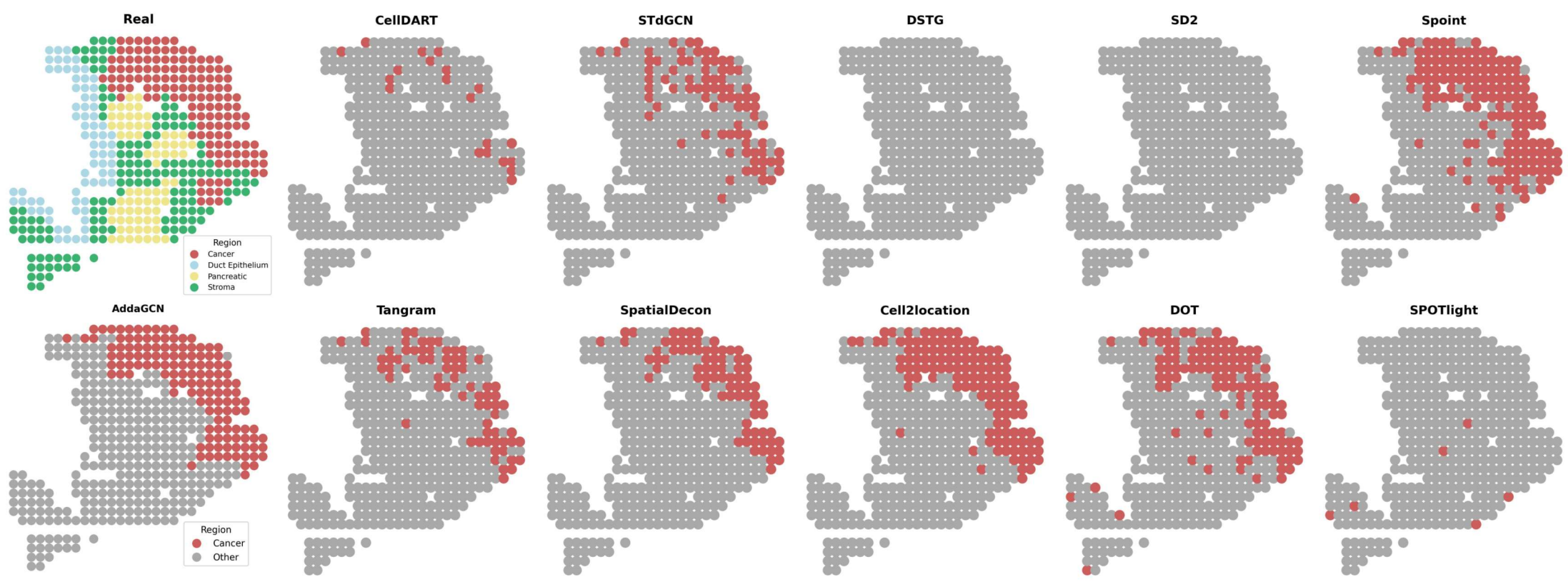

Fig I. Prediction of cancer clone regions by different methods on the PDAC-A section and the cancer regions

annotated in the actual H&E image.

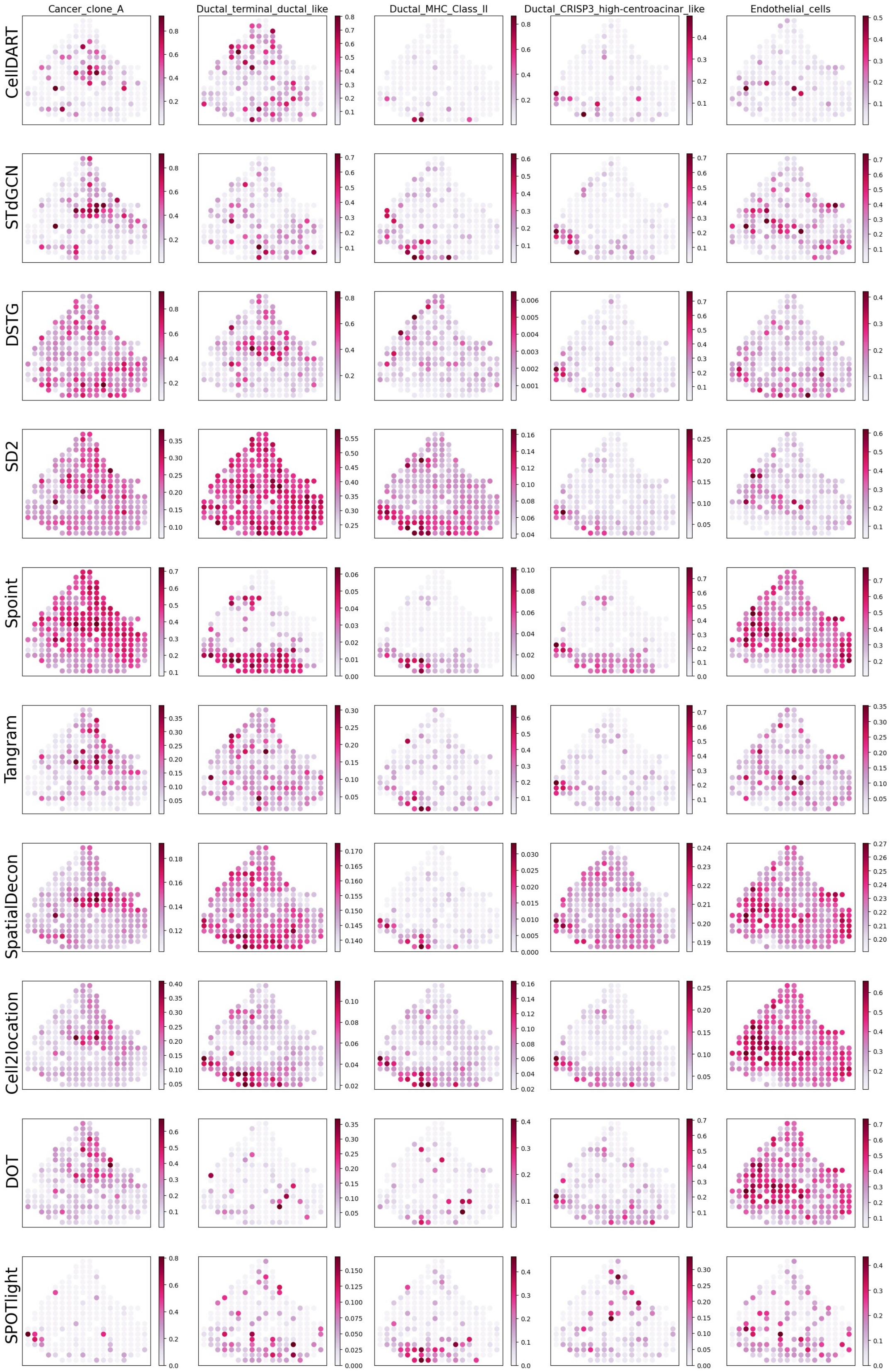

Fig J. The proportion of each of the cell types inferred by different methods is displayed on each spatial location of PDAC-B slice.

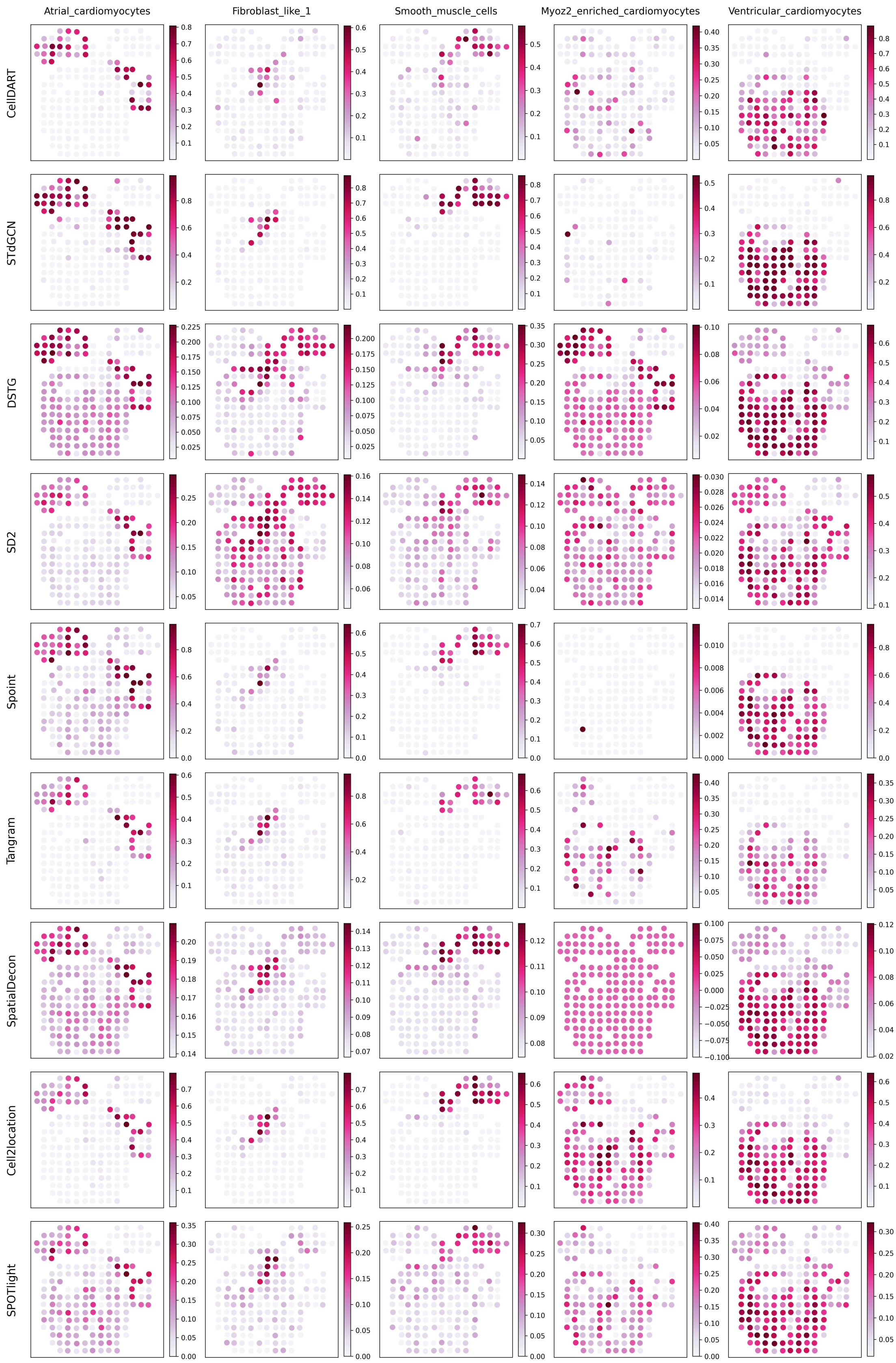

Fig K. Performance of benchmark models in distinguish five celltypes on slice FH6_1000L2_CN74_D1 of the developing human heart spatial transcriptomic dataset.

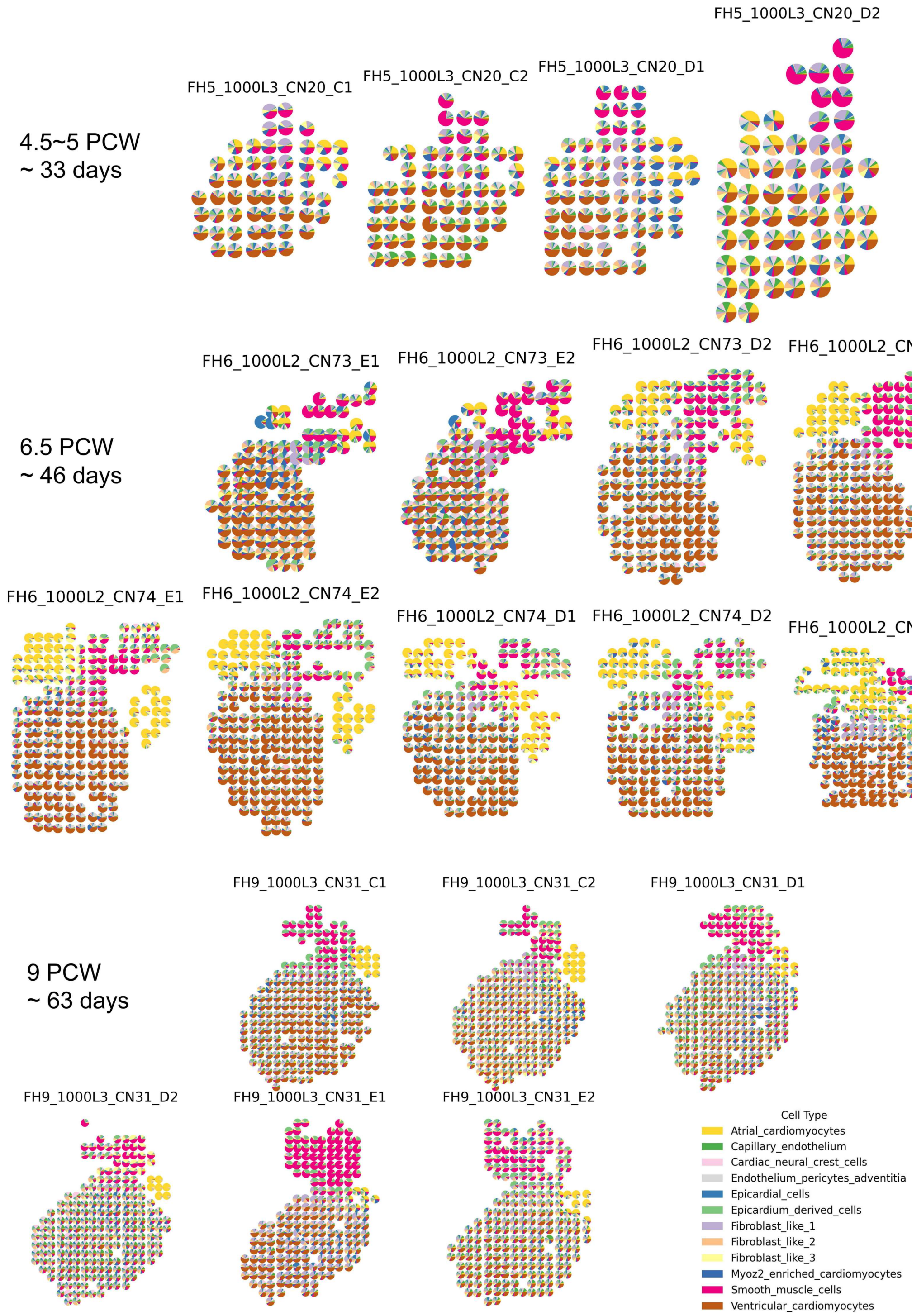

Fig L. Pie plots of the predicted cell type proportions of the developing human heart spatial transcriptomic dataset from AddaGCN.

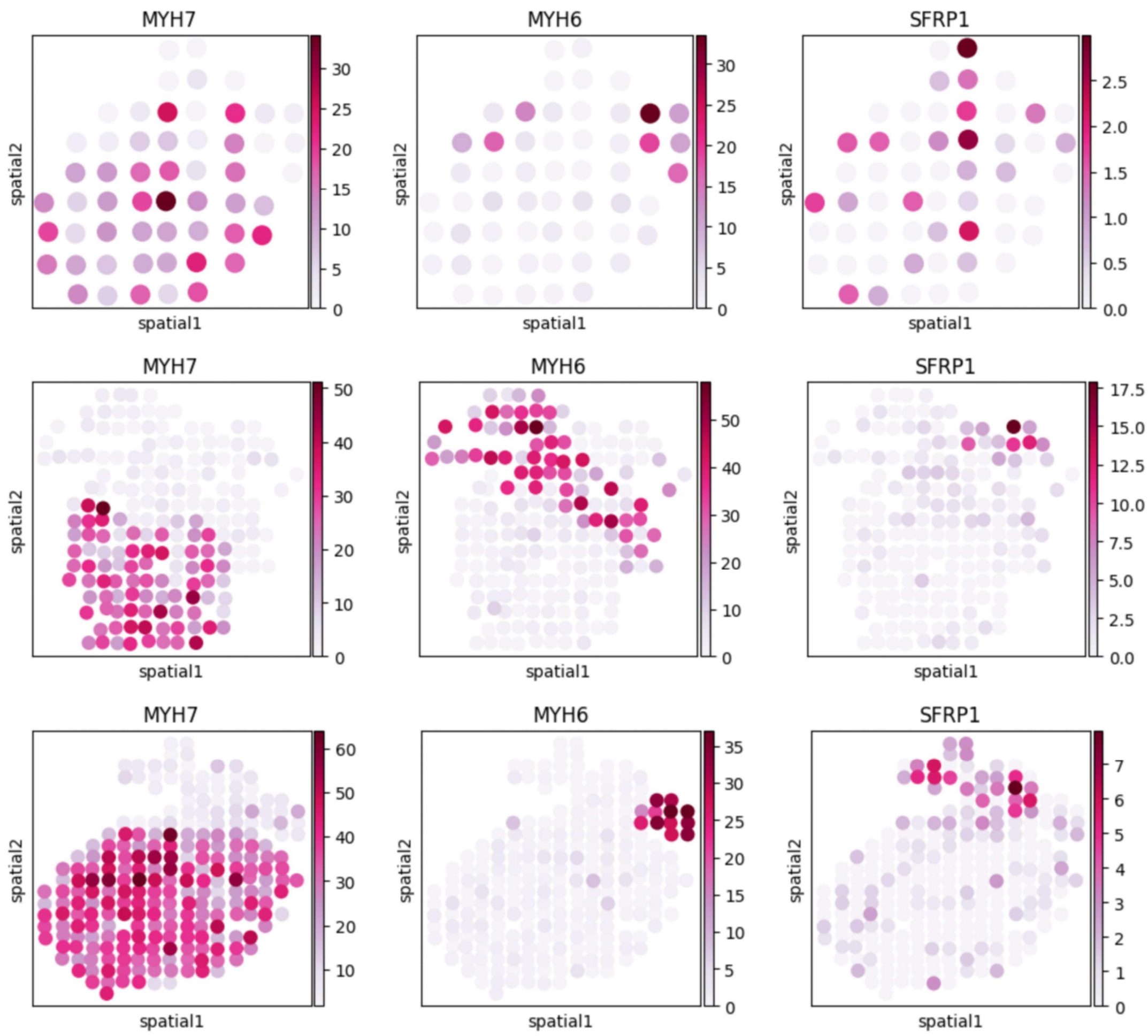

Fig M. The expression levels of corresponding celltype specific marker genes are displayed.
(Ventricular_cardiomyocytes: MYH7; Atrial_cardiomyocytes: MYH6; Smooth_muscle_cells: SFRP1)

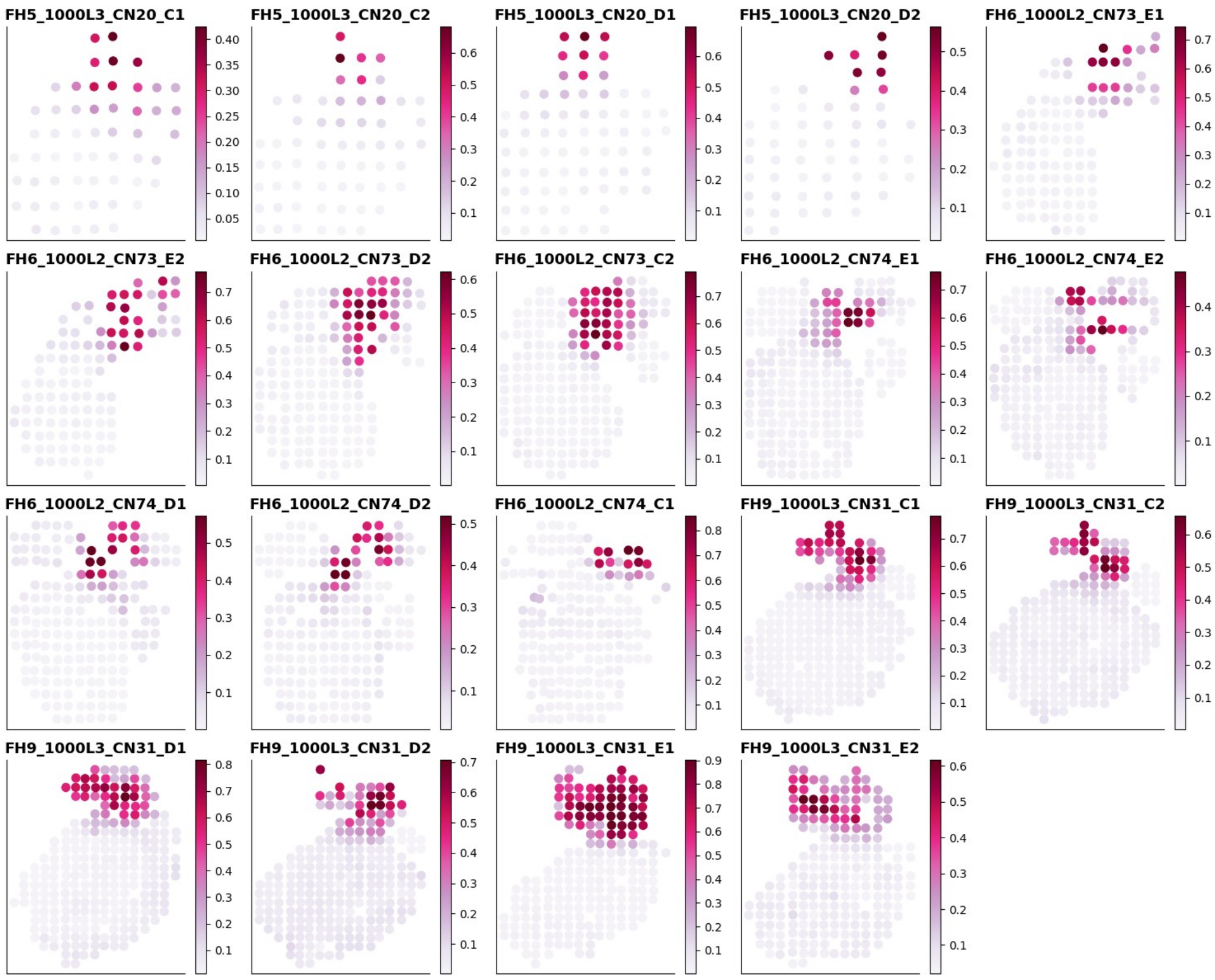

Fig N. The inferred proportions of smooth muscle cells at each spatial position using AddaGCN on the developing human heart ST dataset (FH5: PCW 4.5 – 5; FH6: PCW 6.5; FH9: PCW 9).

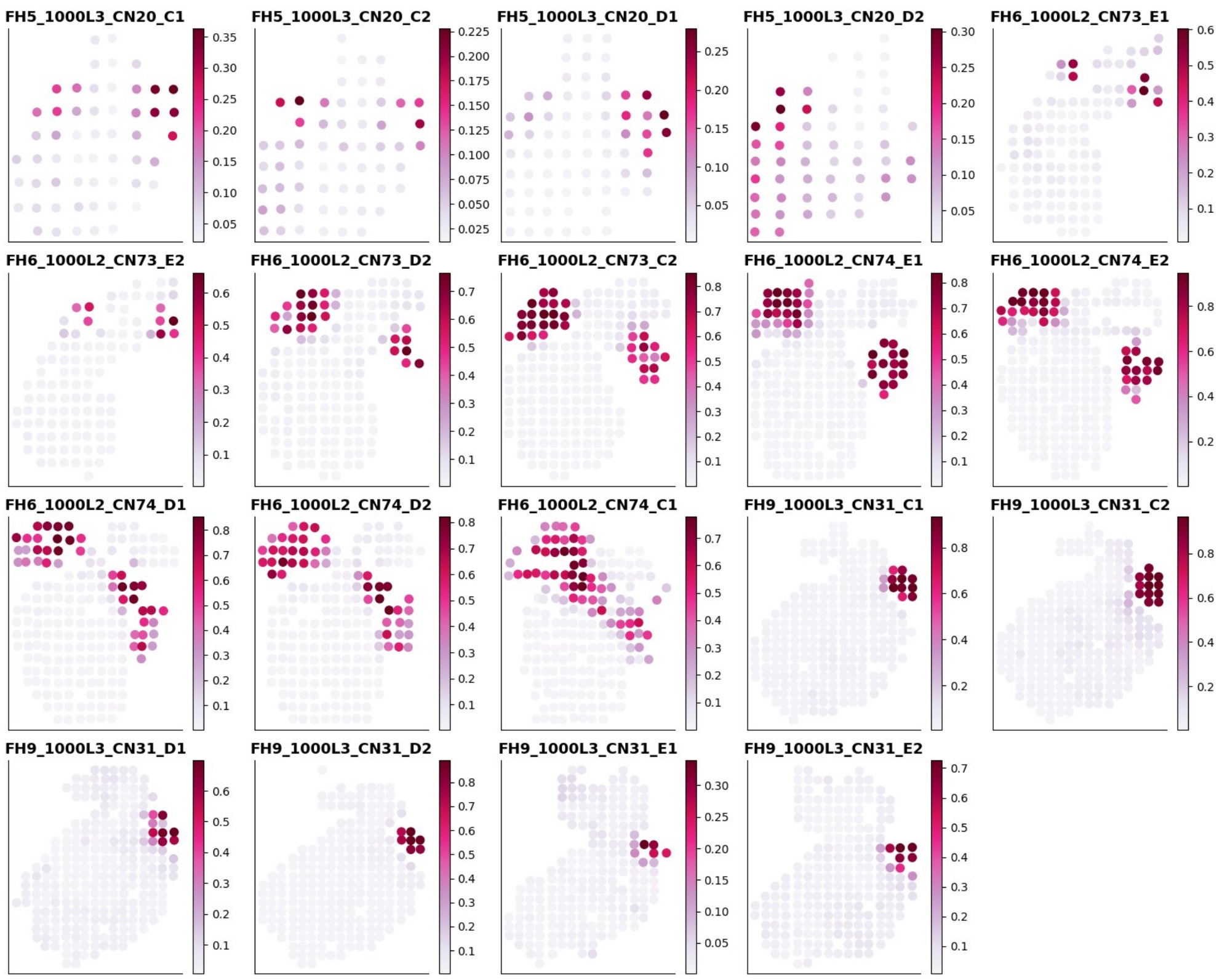

Fig O. The inferred proportions of atrial cardiomyocytes at each spatial position using AddaGCN on the developing human heart ST dataset (FH5: PCW 4.5 – 5; FH6: PCW 6.5; FH9: PCW 9).

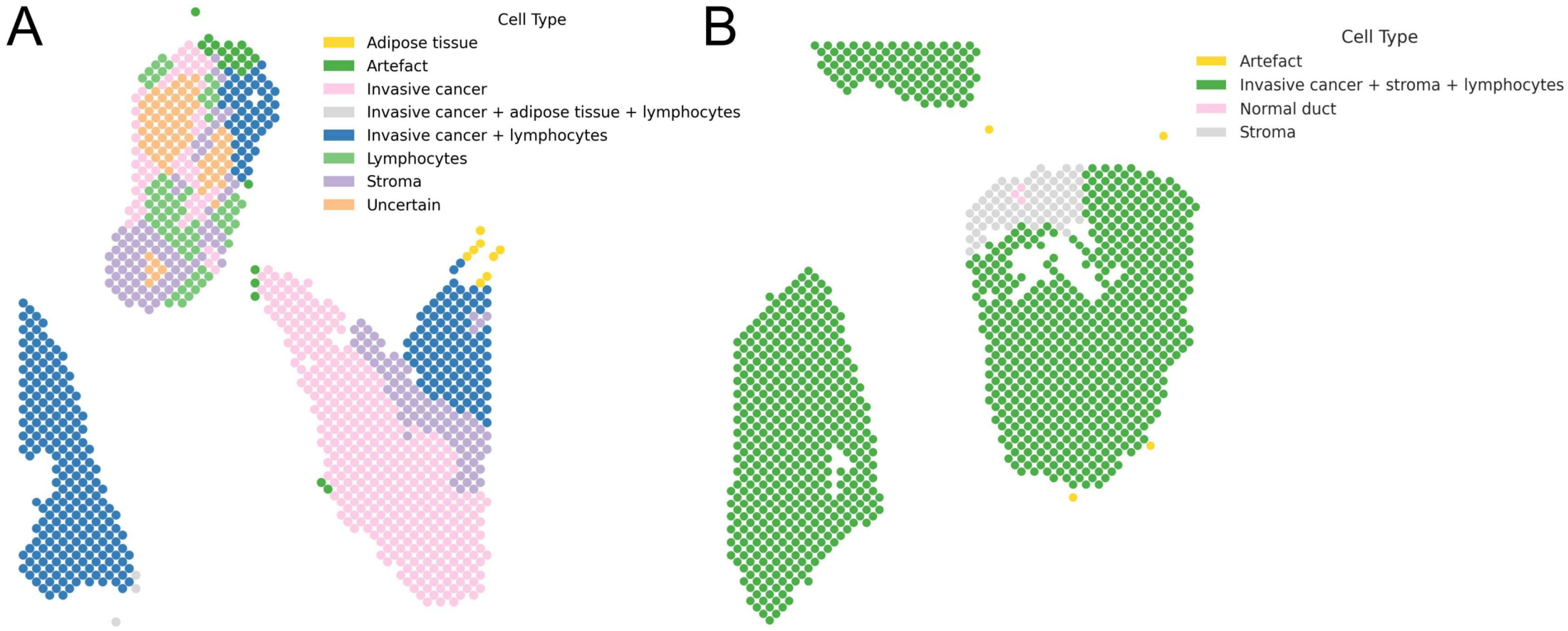

Fig P. Real label annotation information of human breast cancer data slice CID4535 (A) and slice CID4465 (B).

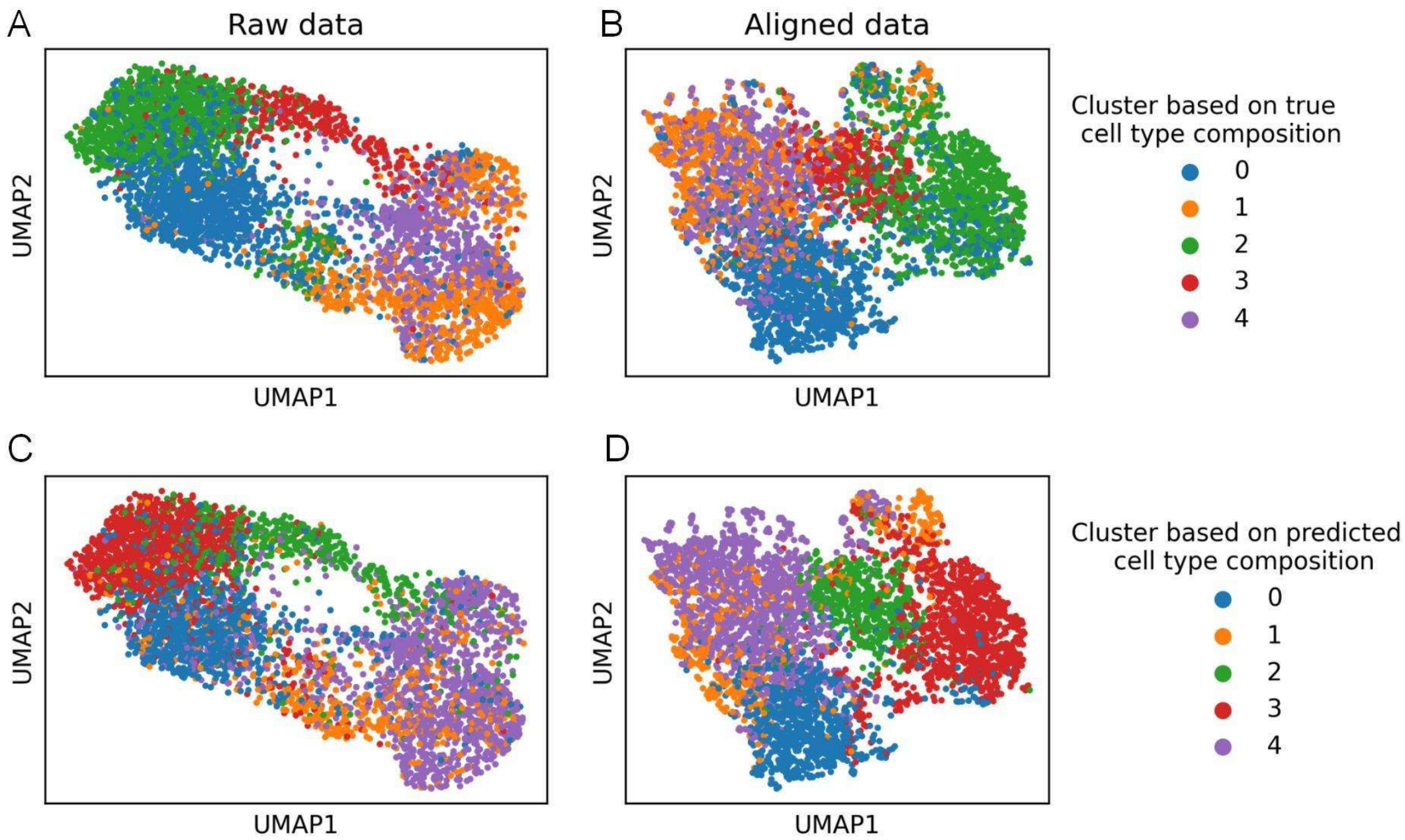

Fig Q. UMAP visualization comparing the consistency between ground-truth labels and deconvolution-predicted clustering results. (A, C) UMAP embeddings of raw expression data, colored by ground-truth cell clusters (A) and deconvolution-predicted clusters (C), respectively; (B, D) UMAP visualization of integrated low-dimensional representations, similarly colored by ground-truth cell clusters (B) and predicted clusters (D). Both ground-truth and deconvolution-predicted cluster assignments were derived from K-means clustering (k = 5) performed on PCA embeddings of the ground-truth and predicted cell-type proportion matrices, respectively.

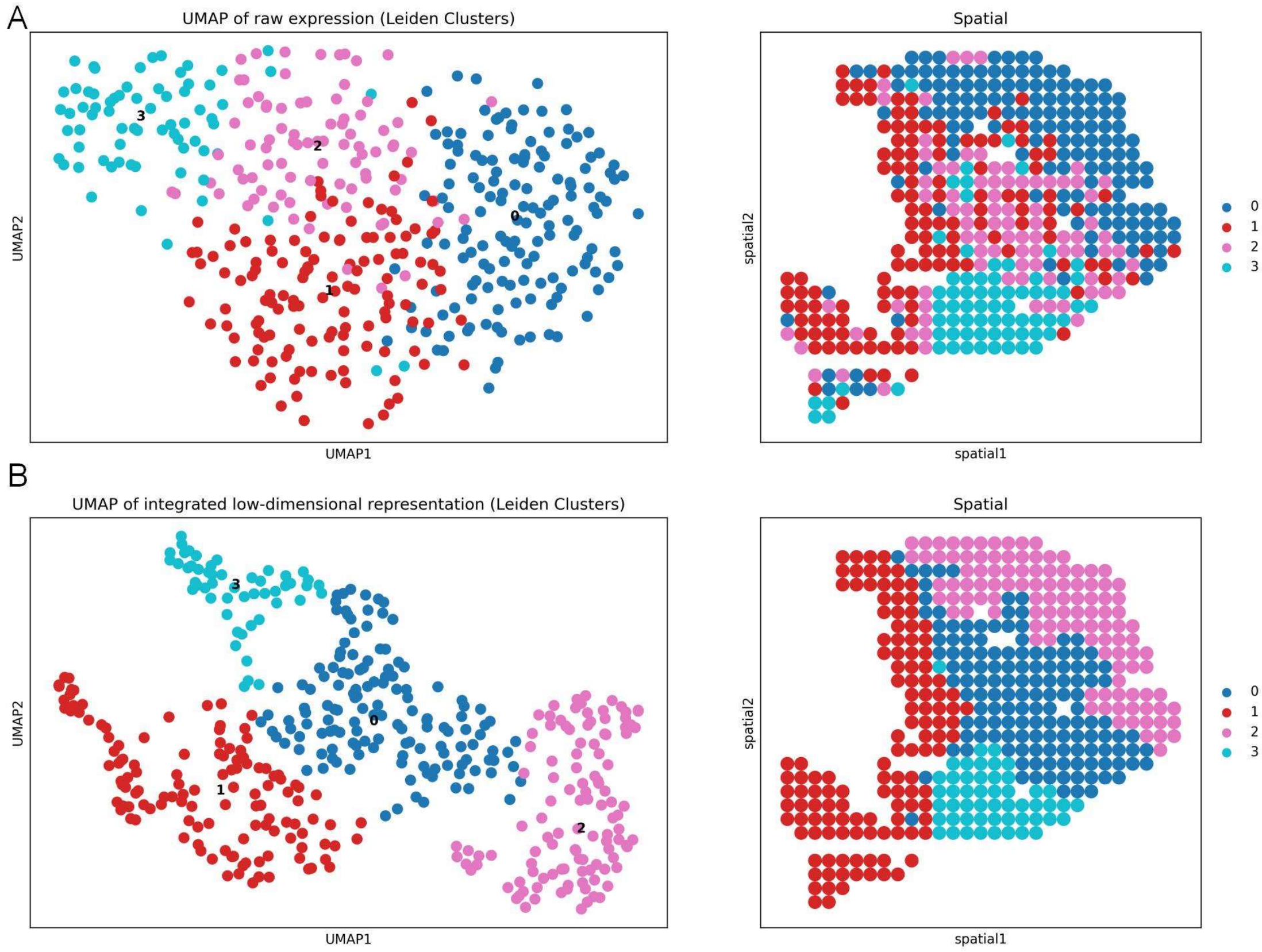

Fig R. Preservation of cell-type structure after alignment. (A) UMAP visualization (left) and spatial distribution (right) of the PDAC-A slice based on raw expression data, with cells colored by Leiden clusters (k=4); (B) UMAP visualization (left) and spatial distribution (right) of the PDAC-A slice based on the integrated low-dimensional representation, with cells colored by Leiden clusters (k=4).

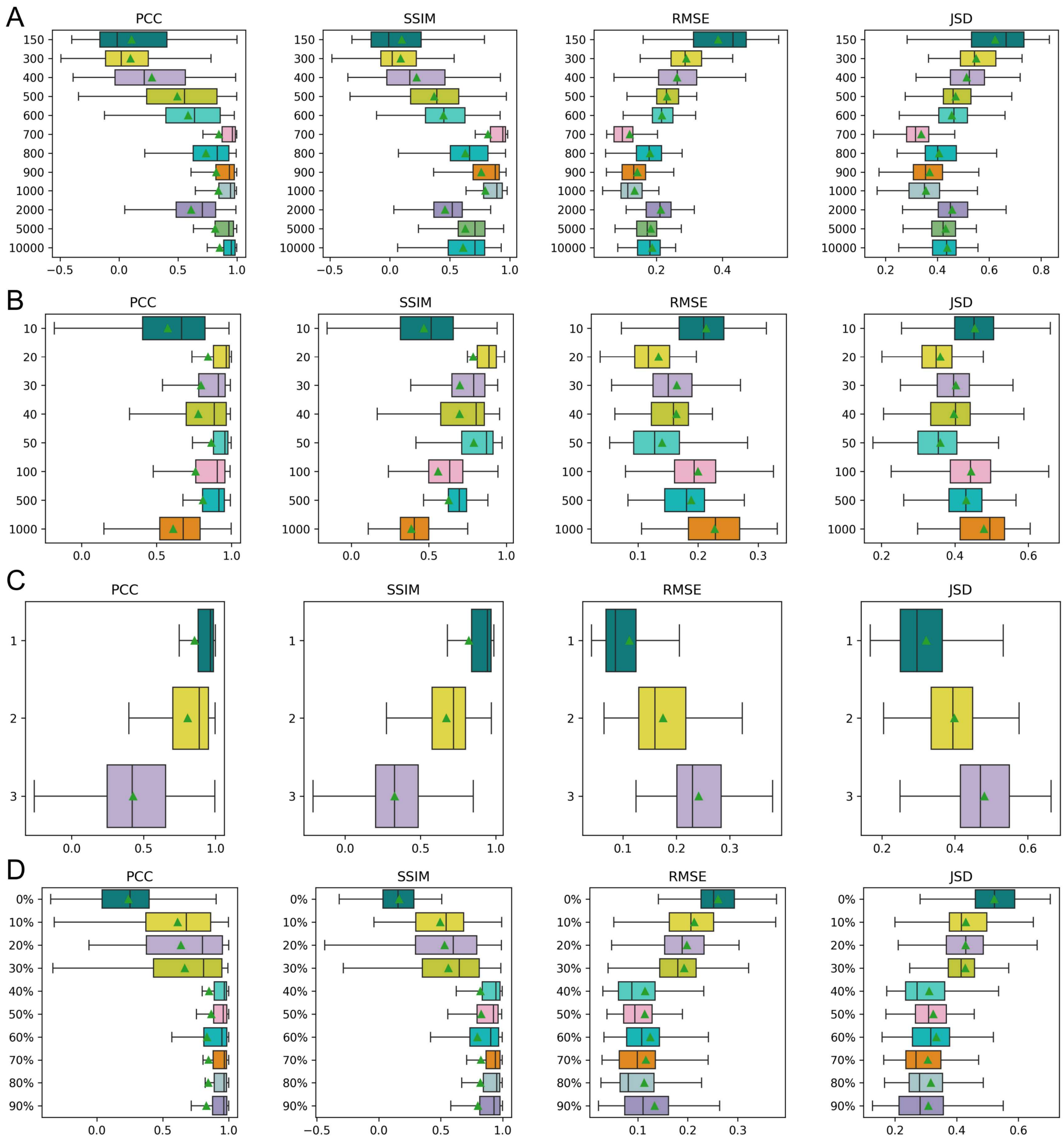

Fig S. Hyperparameter Sensitivity Analysis on the seqFISH+ data. (A-D) Boxplots of PCC, SSIM, RMSE and JSD of each deconvolution method in predicting the cell type distribution of spots. Center line: median; box limits: upper and lower quartiles; whisker: 1.5 × interquartile range; green triangle: mean value; number of predicted spots: 71. Higher PCC and SSIM, lower RMSE and JSD indicate better performance. The number of pseudo spots (A); The number of marker genes in each cell type (B); The fix cell type number of each pseudo spot (C); Proportion of fixed cell type in pseudo spots (D).

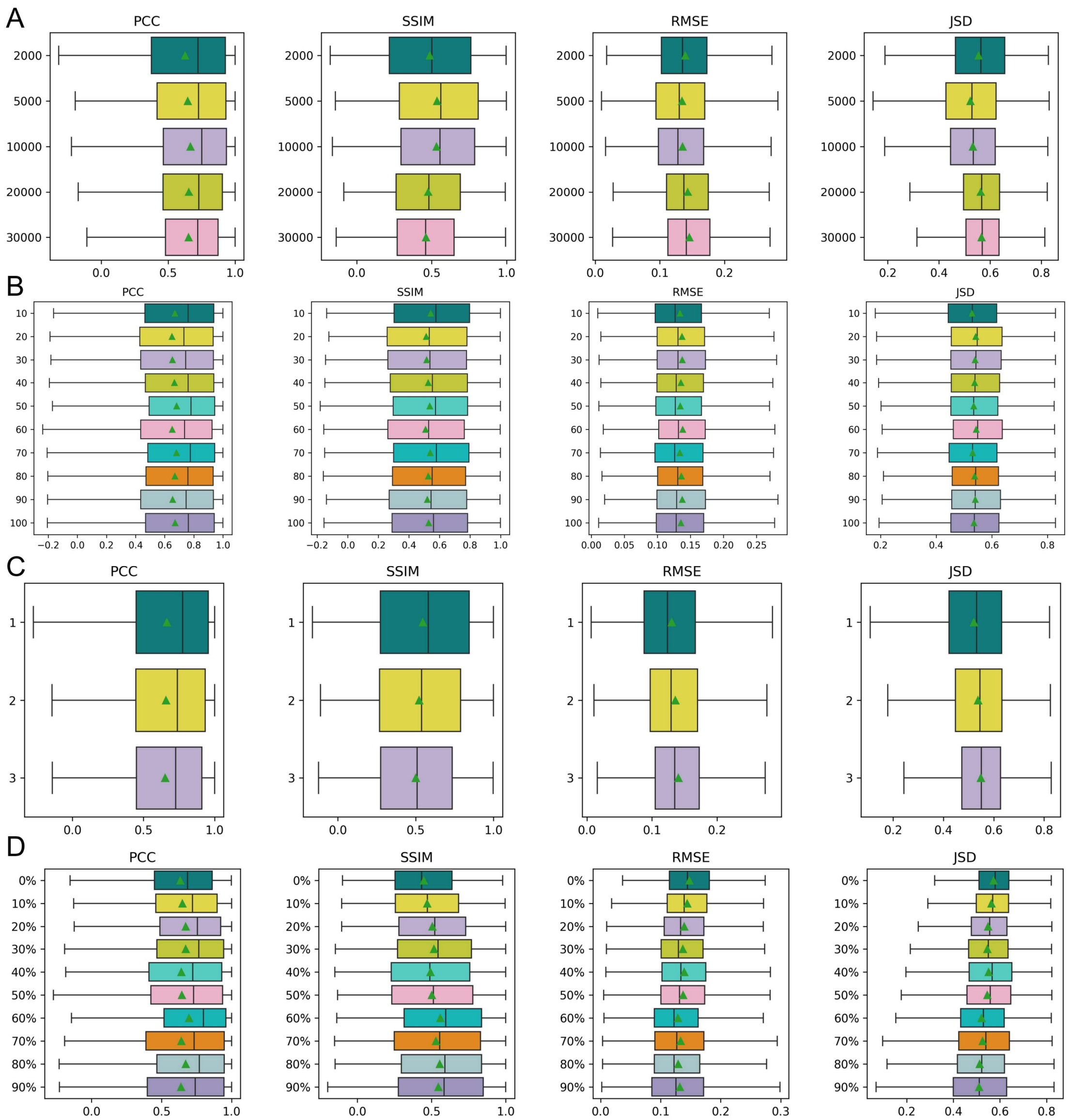

Fig T. Hyperparameter Sensitivity Analysis on the Xenium data. (A-D) Boxplots of PCC, SSIM, RMSE and JSD of each deconvolution method in predicting the cell type distribution of spots. Center line: median; box limits: upper and lower quartiles; whisker: 1.5 × interquartile range; green triangle: mean value; number of predicted spots: 4237. Higher PCC and SSIM, lower RMSE and JSD indicate better performance. The number of pseudo spots (A); The number of marker genes in each cell type (B); The fix cell type number of each pseudo spot (C); Proportion of fixed cell type in pseudo spots (D).

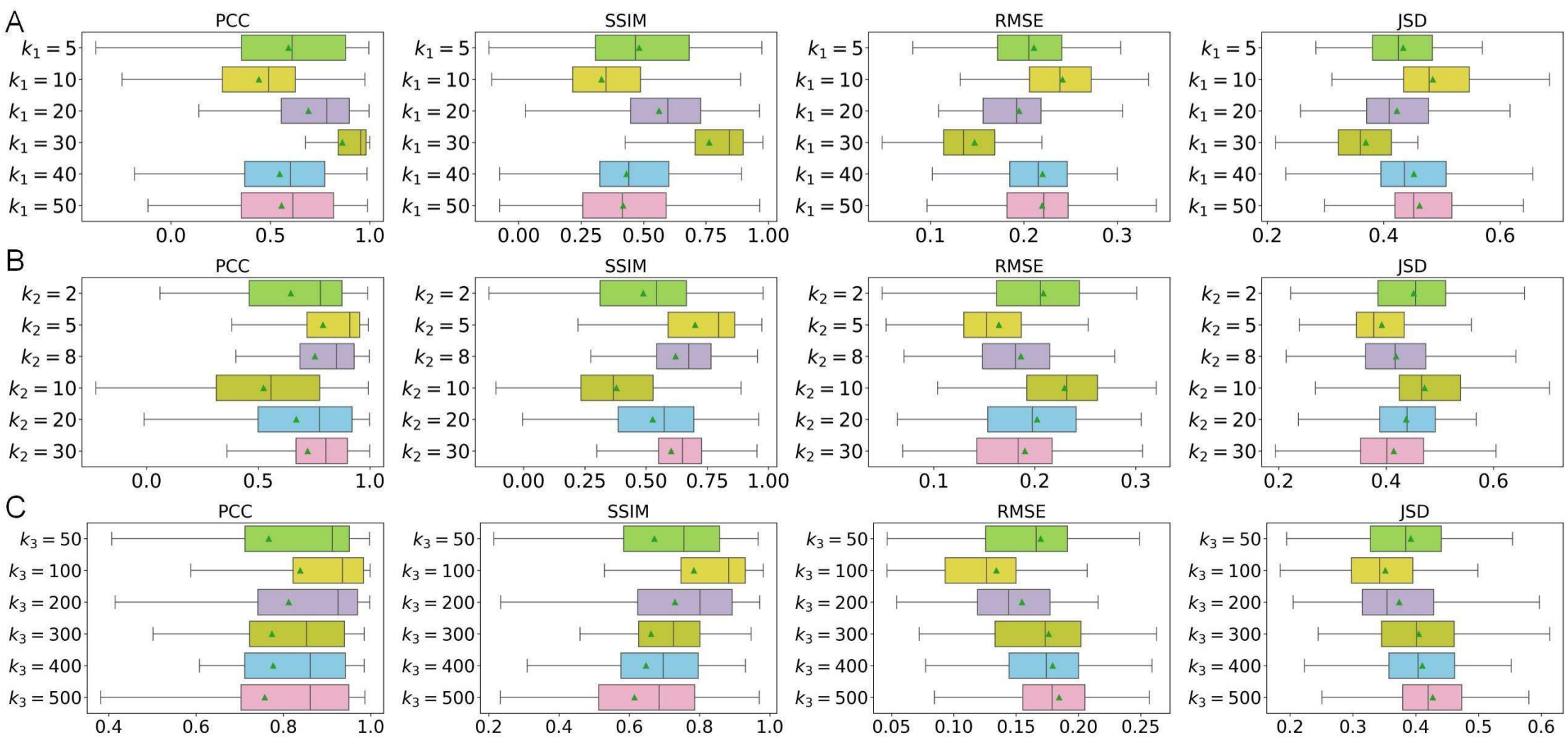

Fig U. Sensitivity analysis results of three key hyperparameters in the AddaGCN graph construction process, including the number of nearest neighbors k_1 (A), the number of mutual nearest neighbor pairs for screening k_2 (B), and the edge filtering threshold in expression space k_3 (C).

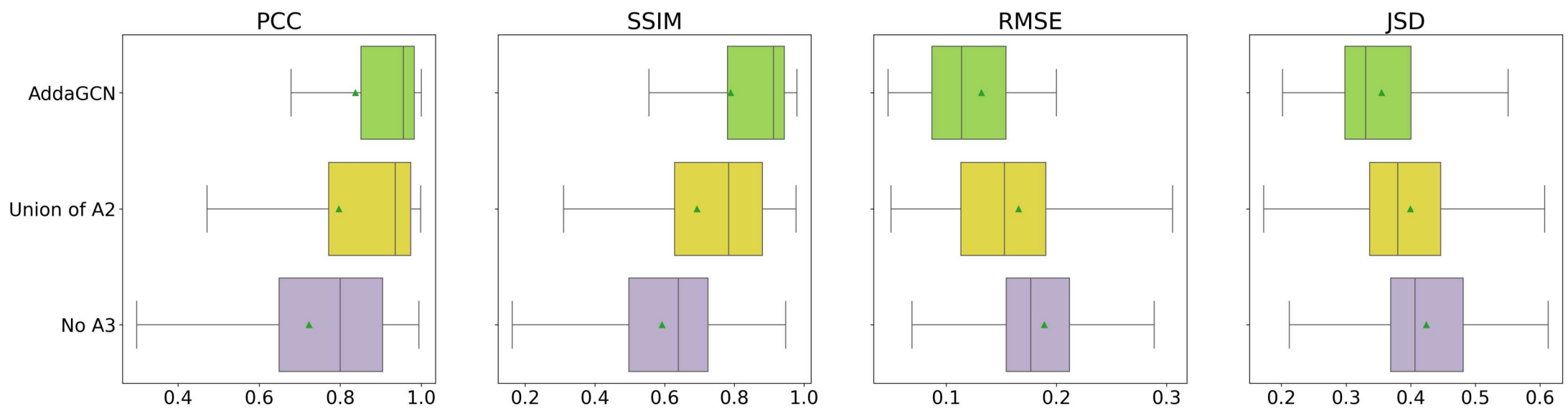

Fig V. The ablation study on the seqFISH+ data comparing the original AddaGCN and the other two conditions: union of A2 (use the union of MNN and SNN to construct real-real graph), no A3 (removing cross-domain connections). Higher PCC and SSIM, lower RMSE and JSD indicate better performance; b, The true and inferred proportions of six major cell types at each spatial position using different conditions on the seqFISH+ simulated data.

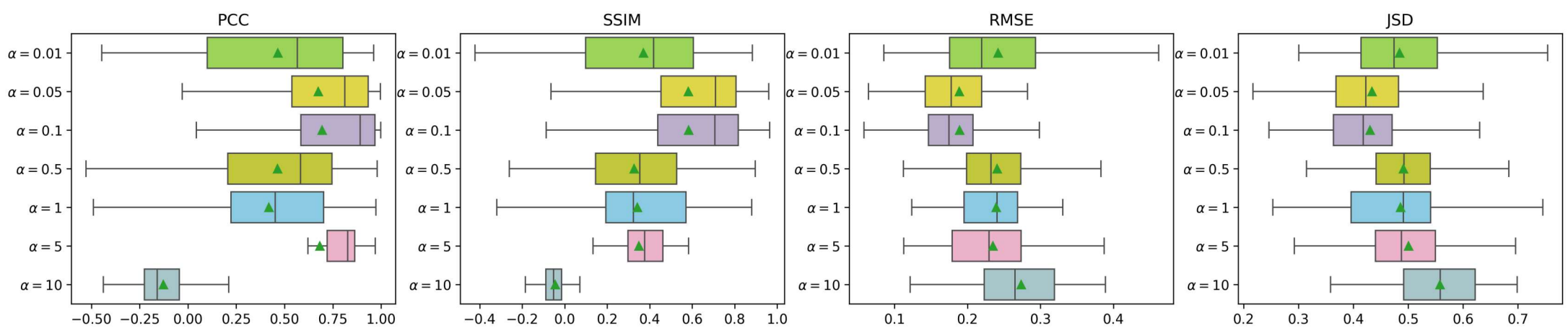

Fig W. Sensitivity analysis of α on the seqFISH+ data. Boxplots of PCC, SSIM, RMSE and JSD of AddaGCN with different α in predicting the cell type distribution of spots.

Reference

[1] Eng CHL, Lawson M, Zhu Q, Dries R, Koulena N, Takei Y, et al. Transcriptome-scale super-resolved imaging in tissues by RNA seqFISH+. Nature. 2019;568(7751):235–9.

[2] Zeisel A, Muñoz-Manchado AB, Codeluppi S, Lönnerberg P, La Manno G, Juréus A, et al. Cell types in the mouse cortex and hippocampus revealed by single-cell RNA-seq. Science. 2015;347(6226):1138–42.

[3] Janesick A, Shelansky R, Gottscho AD, Wagner F, Williams SR, Rouault M, et al. High resolution mapping of the tumor microenvironment using integrated single-cell, spatial and in situ analysis. Nat Commun. 2023;14(1):8353.

[4] Wu SZ, Al-Eryani G, Roden DL, Junankar S, Harvey K, Andersson A, et al. A single-cell and spatially resolved atlas of human breast cancers. Nat Genet. 2021;53(9):1334–47.

[5] Li B, Zhang W, Guo C, Xu H, Li L, Fang M, et al. Benchmarking spatial and single-cell transcriptomics integration methods for transcript distribution prediction and cell type deconvolution. Nat Methods. 2022;19(6):662–70.

[6] Kingma D P, Ba J. Adam: A method for stochastic optimization[J]. arXiv preprint arXiv:1412.6980, 2014.
